# Neural correlates of disaster-related prenatal maternal stress in young adults from Project Ice Storm: Focus on amygdala, hippocampus, and prefrontal cortex

**DOI:** 10.3389/fnhum.2023.1094039

**Published:** 2023-02-01

**Authors:** Xinyuan Li, Muhammad Naveed Iqbal Qureshi, David P. Laplante, Guillaume Elgbeili, Sherri Lee Jones, Suzanne King, Pedro Rosa-Neto

**Affiliations:** ^1^Integrated Program in Neuroscience, McGill University, Montreal, QC, Canada; ^2^Mental Health and Society Division, Douglas Mental Health University Institute, Montreal, QC, Canada; ^3^Translational Neuroimaging Laboratory, McGill University Research Centre for Studies in Aging, Montreal, QC, Canada; ^4^Montreal Neurological Institute, McGill University, Montreal, QC, Canada; ^5^Centre for Child Development and Mental Health, Lady Davis Institute-Jewish General Hospital, Montreal, QC, Canada; ^6^Department of Psychiatry, McGill University, Montreal, QC, Canada; ^7^Department of Neurology and Neurosurgery, McGill University, Montreal, QC, Canada

**Keywords:** volume, resting-state functional connectivity, prenatal maternal stress, amygdala nuclei, hippocampal subfields, prefrontal cortex

## Abstract

**Background:**

Studies have shown that prenatal maternal stress alters volumes of the amygdala and hippocampus, and alters functional connectivity between the amygdala and prefrontal cortex. However, it remains unclear whether prenatal maternal stress (PNMS) affects volumes and functional connectivity of these structures at their subdivision levels.

**Methods:**

T1-weighted MRI and resting-state functional MRI were obtained from 19-year-old young adult offspring with (*n* = 39, 18 male) and without (*n* = 65, 30 male) exposure to PNMS deriving from the 1998 ice storm. Volumes of amygdala nuclei, hippocampal subfields and prefrontal subregions were computed, and seed-to-seed functional connectivity analyses were conducted.

**Results:**

Compared to controls, young adult offspring exposed to disaster-related PNMS had larger volumes of bilateral whole amygdala, driven by the lateral, basal, central, medial, cortical, accessory basal nuclei, and corticoamygdaloid transition; larger volumes of bilateral whole hippocampus, driven by the CA1, HATA, molecular layer, fissure, tail, CA3, CA4, and DG; and larger volume of the prefrontal cortex, driven by the left superior frontal. Inversely, young adult offspring exposed to disaster-related PNMS had lower functional connectivity between the whole amygdala and the prefrontal cortex (driven by bilateral frontal poles, the left superior frontal and left caudal middle frontal); and lower functional connectivity between the hippocampal tail and the prefrontal cortex (driven by the left lateral orbitofrontal).

**Conclusion:**

These results suggest the possibility that effects of disaster-related PNMS on structure and function of subdivisions of offspring amygdala, hippocampus and prefrontal cortex could persist into young adulthood.

## 1. Introduction

The fetal programming hypothesis proposes that fetal exposure to an adverse intrauterine environment induces long-lasting changes in the offspring (Barker, 2003). Studies have shown that pre-natal maternal depression (Li et al., 2022) and disaster-related prenatal maternal stress (PNMS) (Lafortune et al., 2021) are associated with offspring cognitive, socio-emotional and behavioral development that may persist into young adulthood. This hypothesis also applies to brain development (Lautarescu et al., 2020). A recent review suggests that pre-natal maternal depression, anxiety and stressful life events are associated with atypical volumes and functional connectivity of widespread brain regions in the offspring (Lautarescu et al., 2020).

Prenatal maternal stress has been associated with heightened stress reactivity in the offspring (Yong Ping et al., 2015), which may be explained by alterations in the development of the fetal brain. In the brain, the amygdala, hippocampus and prefrontal cortex contain a very high density of glucocorticoid receptors, and are thus highly sensitive to elevated levels of glucocorticoids (Herman et al., 2005). The amygdala, hippocampus and prefrontal cortex are implicated in the regulation of the hypothalamic pituitary adrenal (HPA) axis: the amygdala is implicated in activation of glucocorticoid secretion whereas the hippocampus and prefrontal cortex are largely inhibitory to glucocorticoid secretion (Herman et al., 2005). It has been documented that the amygdala, hippocampus and prefrontal cortex are concurrently vulnerable to stress exposure (McEwen et al., 2016; Henigsberg et al., 2019; Merino et al., 2021). Moreover, the three regions have been reported to work in concert to regulate the stress response: the amygdala detects potential danger; the hippocampus encodes environmental information associated with the stressor and the prefrontal cortex modulates associations between cues and stressor (Godoy et al., 2018).

The amygdala is a key structure involved in the regulation of fear (Ressler, 2010). Structural magnetic resonance imaging (MRI) research has shown that pre-natal maternal cortisol (Buss et al., 2012), depression (Wen et al., 2017), anxiety (Acosta et al., 2019), and disaster-related PNMS (Jones et al., 2019) are associated with increased whole amygdala volume in young children (Buss et al., 2012; Wen et al., 2017; Acosta et al., 2019) and in adolescents (Jones et al., 2019). The amygdala, however, is a heterogeneous structure composed of multiple nuclei that play distinct functional roles (Sah et al., 2003). For instance, the lateral and basal nuclei are involved in fear acquisition, and the central and medial nuclei are involved in fear expression and execution of fear responses (Ressler, 2010; Duvarci and Pare, 2014). To date, there is scant literature on associations between PNMS and offspring amygdala nuclei volumes.

The hippocampus plays a central role in learning and memory (Wingenfeld and Wolf, 2014). Three studies have shown that pre-natal maternal psychological distress (Lehtola et al., 2020; Moog et al., 2021) and anxiety (Wu et al., 2020) are associated with decreased whole hippocampal volume in fetuses (Wu et al., 2020) or in neonates (Lehtola et al., 2020; Moog et al., 2021); disaster-related PNMS has been associated with increased whole hippocampal volume in adolescents (Cao-Lei et al., 2021), while two other studies have reported no associations between pre-natal maternal cortisol (Buss et al., 2012) or stressful life events (Marečková et al., 2018) and whole hippocampal volume in children (Buss et al., 2012) or in young adults (Marečková et al., 2018). The mixed findings may be attributed to differences in types of stress and offspring age at scanning. As with the amygdala, the hippocampus has been divided into multiple subfields with distinct functional roles (Fogwe et al., 2018). For example, rodent evidence showed that PNMS was associated with a reduction of offspring dendritic arborization and synaptic density in the CA1 and CA3 subfields but not in others (Moog et al., 2021). There is little research on associations between PNMS and hippocampal subfield volumes in human offspring, however.

There is an emerging literature on PNMS and offspring brain functional connectivity. The prefrontal cortex modulates cognitive control functions and is mainly involved in working memory, self-regulatory, and goal-directed behaviors (McEwen and Morrison, 2013). It may act as a hub system that integrates sensory, affective, social, and memory-related information from the amygdala and the hippocampus to coordinate behavioral and peripheral physiological responses according to contextual demands that are appraised as stressful (Ginty et al., 2019). One study showed that pre-natal maternal anxiety was associated with reduced prefrontal gray matter density in child offspring (Buss et al., 2010).

Resting-state functional MRI studies on humans reported associations between PNMS and offspring whole amygdala-prefrontal resting-state functional connectivity (rs-FC). Specifically, pre-natal stressful life events were associated with decreased whole amygdala-medial prefrontal rs-FC in infants (Humphreys et al., 2020). Pre-natal maternal depression was associated with increased rs-FC from the whole amygdala to the anterior cingulate cortex and orbitofrontal cortex in infants (Qiu et al., 2015). Pre-natal maternal depression was associated with increased rs-FC from the whole amygdala to the ventromedial (Qiu et al., 2015) and the dorsal prefrontal cortex (Posner et al., 2016) in infants. The discrepancies noted here paint a potentially complex picture that depends on types of stress and on prefrontal subregions. Compared to the abundant research on the effects of PNMS on whole amygdala-prefrontal rs-FC, the influence of PNMS on whole hippocampus-prefrontal rs-FC has been much less explored. It remains unclear on associations between PNMS and functional connectivity of amygdala nuclei-prefrontal subregions and hippocampal subfields-prefrontal subregions.

In contrast to pre-natal maternal depression, anxiety, and stressful life events, studying PNMS deriving from sudden-onset natural disasters has several advantages, primarily because natural disasters are independent events that are not confounded by parental characteristics. In January 1998, five continuous days of freezing rain produced an ice storm in southern Quebec, Canada, that resulted in the failure of the regional power grid depriving millions of residents of electricity. In June 1998, we launched the world’s first prospective longitudinal disaster-related PNMS cohort: Project Ice Storm. This project recruited women who were pregnant during the crisis or became pregnant within 3 months following the ice storm, and comprehensively assessed three aspects of PNMS in each woman: objective hardship, subjective distress and cognitive appraisal of the crisis. The measurements of Project Ice Storm offspring began from age 6 months, and continued approximately every 2 years thereafter, till 19 years old.

Here, we studied 19-year-old young adult offspring from Project Ice Storm and typically developing controls from two datasets: the Autism Brain Imaging Data Exchange (ABIDE) and the Attention Deficit Hyperactivity Disorder-200 (ADHD-200). Our first goal was to examine group differences between young adult offspring exposed to disaster-related PNMS and controls in volumes of amygdala nuclei, hippocampal subfields and prefrontal subregions. Our secondary goal was to examine the group differences in rs-FC of amygdala nuclei-prefrontal subregions and hippocampal subfields-prefrontal subregions. Finally, we aimed to determine the extent to which the severity of disaster-related PNMS was associated with any volume and rs-FC showing between-group differences.

## 2. Materials and methods

### 2.1. Participants

#### 2.1.1. Ice Storm

In June 1998, the initial Project Ice Storm cohort consisted of 176 women. At age 19, there were 39 young adult offspring (18M/21F) who underwent structural MRI and resting-state functional MRI scans. As shown in Supplementary Table 1, no significant differences were detected on the three aspects of PNMS between the current sample of 39 families and the 137 families who did not participate in MRI scanning at age 19; the difference was that the current sample of 39 families had significantly higher socioeconomic status. Among the 39 mothers, 30.8% (12/39) were middle class; 46.2% (18/39) were upper middle class; and 23.1% (9/39) were upper class. When the ice storm peaked on 9 January 1998, 28.2% (11/39) were within 3-month of conception; 30.8% (12/39) were in the 1^st^ trimester of pregnancy; 23.1% (9/39) were in the 2^nd^ trimester; and 17.9% (7/39) were in the 3^rd^ trimester.

All phases of Project Ice Storm were approved by the Research Ethics Board of Douglas Mental Health University Institute. All the participants provided written informed consent.

#### 2.1.2. Controls

Control participants were obtained from the ABIDE^1^ and the ADHD-200^2^. All control participants were typically developing participants who were characterized by absence of Autism Spectrum Disorder and Attention-Deficit/Hyperactivity Disorder diagnoses, as well as by absence of major neurological or psychiatric disorders. Despite no measurements of PNMS in controls, no natural disasters were recorded between 1996 and 1999 in the locations where the participants were recruited. As such, we can assume that the control participants were not systematically exposed to a population-level natural disaster. Control participants were selected to match Ice Storm participants on age, sex, handedness, intelligence quotient and in-scanner head motion (Supplementary Table 2).

### 2.2. Three aspects of prenatal maternal stress

#### 2.2.1. Objective hardship

In June 1998, 5 months after the onset of the ice storm, the severity of maternal objective hardship experienced by pregnant women was assessed according to four dimensions of disaster exposure: Threat (e.g., injuries), Loss (e.g., loss of personal income), Scope (e.g., duration without electricity), and Change (e.g., temporary shelter) (Bromet and Dew, 1995; Laplante et al., 2007). Each dimension was scored on a scale of 0 (no exposure) to 8 (high exposure). A total score, referred to as Storm32, was calculated by summing scores across all four dimensions (Laplante et al., 2007). The test-retest reliability of Storm32 (assessed in the same women 6 years later) was satisfactory (*r* = 0.79) (St-Hilaire et al., 2015).

#### 2.2.2. Subjective distress

In June 1998, maternal subjective distress was assessed using a validated 22-item French version (Brunet et al., 2003) of the Impact of Event Scale–Revised (IES-R) (Weiss et al., 1997), the gold-standard screening for post-traumatic stress disorder (PTSD). A total score of 33 is a cut-off for probable PTSD (Creamer et al., 2003). The scale rates the severity of symptoms in the preceding 7 days in three dimensions relevant to PTSD: intrusive thoughts, hyperarousal, and avoidance. Each dimension was scored of 0 (not at all) to 4 (extremely). The scale has satisfactory test-retest reliability for the total score (*r* = 0.76) (Brunet et al., 2003). Log-transformed values of the total score were used in the analyses due to skewed distribution.

#### 2.2.3. Cognitive appraisal

In June 1998, maternal cognitive appraisal of the crisis was assessed using the following question: “Overall, what were the consequences of the ice storm on you and your family?.” Response options were rated as three options: “negative” (“–1”), “neutral” (“0”), and “positive” (“1”). We have shown that this measure has predictive validity by significantly correlating with child outcomes [e.g., BMI and central adiposity (Cao-Lei et al., 2016), C-peptide (Cao-Lei et al., 2018), and DNA methylation (Cao-Lei et al., 2015)].

### 2.3. MRI data acquisition

#### 2.3.1. Ice Storm

MR images were acquired using a 3T Siemens MAGNETOM Trio TIM Syngo MRI scanner, with a 12-channel head coil. Anatomical images were obtained using a T1-weighted (T1w) Magnetization Prepared Rapid Gradient Echo sequence: 192 slices; Repetition Time (TR) = 2,400 s; Echo Time (TE) = 2.43 ms; slice thickness = 1 mm; flip angle = 8°; matrix = 256 × 256. Resting-sate functional images were acquired using a T2*-weighted echo-planar imaging sequence: 42 slices; TR = 2,600 ms; TE = 30 ms; flip angle = 90°; slice thickness = 3.4 mm; FoV = 218 mm, matrix = 64 × 64. Throughout the 5:01 min resting-state functional MRI scan, participants were instructed to lie still with their eyes open.

#### 2.3.2. Controls

The scanning parameters of controls are described in Supplementary Table 3.

### 2.4. MRI data pre-processing

One Ice Storm participant was excluded from resting-state functional MRI pre-processing due to scan artifacts (Supplementary Figure 1). fMRIPrep 1.5.7 (Esteban et al., 2019) was used for pre-processing. The T1w image was corrected for intensity non-uniformity with N4BiasFieldCorrection (Tustison et al., 2010), distributed with ANTs 2.2.0 (Avants et al., 2008), and used as T1w-reference throughout the workflow. The T1w-reference was then skull-stripped with a Nipype implementation of the antsBrainExtraction.sh workflow (from ANTs), using OASIS30ANTs as target template. Brain tissue segmentation of gray matter, white matter and cerebrospinal fluid was performed on the brain-extracted T1w using fast FSL 5.0.9 (Zhang et al., 2001). Volume-based spatial normalization to the Montreal Neurological Institute (MNI) space was performed through non-linear registration with ants. Registration (ANTs 2.2.0), using brain-extracted versions of both T1w reference and the T1w template. For each of the blood-oxygen-level-dependent (BOLD) runs found per subject, the following pre-processing was performed. First, a reference volume and its skull-stripped version were generated using a custom methodology of fMRIPrep. A deformation field to correct for susceptibility distortions was estimated based on fMRIPrep’s fieldmap-less approach. The deformation field is that resulting from co-registering the BOLD reference to the same-subject T1w-reference with its intensity inverted (Huntenburg, 2014; Wang et al., 2017). Registration is performed with ants. Registration (ANTs 2.2.0), and the process regularized by constraining deformation to be non-zero only along the phase-encoding direction, and modulated with an average fieldmap template (Treiber et al., 2016). Based on the estimated susceptibility distortion, a corrected echo-planar imaging reference was calculated for a more accurate co-registration with the anatomical reference. The BOLD reference was then co-registered to the T1w reference using flirt FSL 5.0.9 (Jenkinson and Smith, 2001) with the boundary-based registration (Greve and Fischl, 2009) cost-function. Co-registration was configured with nine degrees of freedom to account for distortions remaining in the BOLD reference. Head-motion parameters with respect to the BOLD reference (transformation matrices, and six corresponding rotation and translation parameters) are estimated before any spatiotemporal filtering using mcflirt FSL 5.0.9 (Jenkinson et al., 2002). The BOLD time-series were resampled onto their original, native space by applying a single, composite transform to correct for head-motion and susceptibility distortions. The BOLD time-series were then resampled into the MNI space. Automatic removal of motion artifacts using independent component analysis (ICA-AROMA) (Pruim et al., 2015), was performed on the spatially normalized, pre-processed BOLD on MNI space time-series after removal of non-steady state volumes and spatial smoothing with an isotropic, Gaussian kernel of 6 mm FWHM. In addition, we conducted white matter and cerebrospinal fluid signal removal from the BOLD time series and temporally bandpass filtering (>0.01 Hz).

### 2.5. FreeSurfer segmentation

**FIGURE 1:**
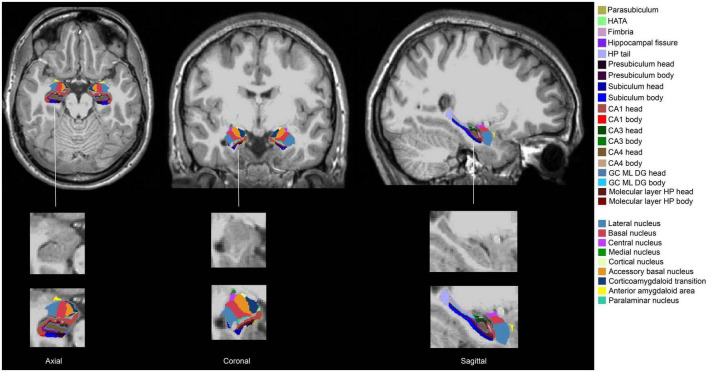
Segmentation of the hippocampus and the amygdala of one Ice Storm participant. The columns from left to right represent axial, coronal, and sagittal views, respectively. The hippocampal subfields and the amygdala nuclei (**left** and **right**) were labeled with different colors. The second-row zooms in the right hippocampus and the right amygdala without any labels as the reference. The third-row zooms in the right hippocampus and the right amygdala with labels as the reference.

**FIGURE 2:**
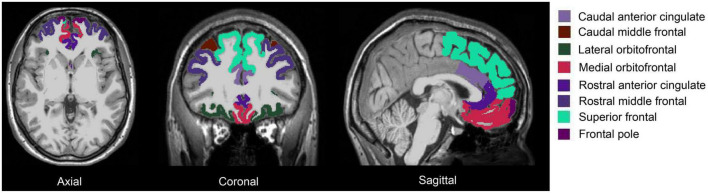
Segmentation of the prefrontal cortex of one Ice Storm participant. The columns from left to right represent axial, coronal, and sagittal views, respectively. The prefrontal subregions (**left** and **right**) were labeled with different colors.

fMRIPrep pre-processed T1w brain regions in MNI space were segmented using FreeSurfer 7.1.1 (Fischl, 2012) and its library tool *recon-all*. The segmentation of the amygdala and the hippocampus was performed using *segmentHA_T1.sh* (Iglesias et al., 2015; Saygin et al., 2017). The amygdala nuclei and hippocampal subfields are shown in Figure 1: there were 9 amygdala nuclei, and 19 hippocampal subfields. AFNI *3dcalc* was used to combine the hippocampal head and body: the 19 subfields were regrouped into 12 subfields. Eight prefrontal subregions were obtained based on the Desikan-Killiany Atlas (Figure 2). The quality of the segmentation was visually inspected with FreeView by X.L. The anterior amygdaloid area was absent for one Ice Storm participant and three controls (Supplementary Figure 2).

### 2.6. Between-group volumetric differences

Using IBM SPSS Statistics 22 (IBM Corp, 2019), an analysis of covariance (ANCOVA) model, controlling for TR, TE, and sex, was conducted to compare Ice Storm participants with controls on volumetric differences of: (1) whole amygdala; (2) whole hippocampus; (3) 9 amygdala nuclei; (4) 12 hippocampal subfields, and (5) 16 prefrontal subregions. The ANCOVA results were false discovery rate (FDR)-corrected for 18 comparisons based on 9 amygdala nuclei (left and right), 24 comparisons based on 12 hippocampal subfields (left and right), and 16 comparisons based on the eight prefrontal subregions (left and right). We used partial eta squared (η^2^_*p*_) as effect sizes (0.01 = small; 0.06 = medium; 0.14 = large) (Cohen, 1988; Lakens, 2013). We conducted sensitivity analyses by omitting the five participants with the 1.5T scanner. The sensitivity analyses indicated that there were no changes to the significance levels in the ANCOVA results.

### 2.7. Seed-to-seed rs-FC and between-group rs-FC differences

At the individual level, using the whole amygdala or whole hippocampus segmented in MNI space (left and right, separately) as the seed, Pearson correlation coefficients were calculated between the average BOLD time courses extracted from the whole amygdala or whole hippocampus and the average BOLD time courses of the prefrontal subregions. Using the 9 amygdala nuclei or 12 hippocampal subfields (left and right, separately) segmented in MNI space as seeds, Pearson correlation coefficients were calculated between the average BOLD time courses extracted from the 9 amygdala nuclei or 12 hippocampal subfields and the average BOLD time courses of the prefrontal subregions. Resultant seed-to-seed Pearson correlation coefficients were converted to normally distributed *z*-values.

Using the CONN functional connectivity toolbox 19b (Whitfield-Gabrieli and Nieto-Castanon, 2012), ANCOVA controlling for TR, TE, in-scanner eye status and sex, was applied to test differences between Ice Storm participants and controls in: (1) whole amygdala-prefrontal rs-FC; (2) whole hippocampus-prefrontal rs-FC; (3) amygdala nuclei-prefrontal rs-FC, and (4) hippocampal subfield-prefrontal rs-FC. Significance thresholds were set to *p* < 0.001 for uncorrected, and *p* < 0.05 for FDR-correction. Due to the segmentation failure of the anterior amygdaloid area, one Ice Storm participant and three controls were excluded from the rs-FC analyses of the whole amygdala and the anterior amygdaloid area. Finally, the ANCOVA results were FDR-corrected for 18 comparisons based on 9 amygdala nuclei (left and right), and 24 comparisons based on 12 hippocampal subfields (left and right). We conducted sensitivity analyses by omitting the five participants with the 1.5T scanner. The sensitivity analyses indicated that there were no changes to the significance levels in the ANCOVA results.

### 2.8. Association between PNMS and volume and rs-FC within the Ice Storm group

For volume and rs-FC showing significant FDR-corrected between-group differences, the following linear regressions within the Ice Storm group were conducted with IBM SPSS Statistics 22 (IBM Corp, 2019), controlling for sex, to determine: (1) associations between the three aspects of PNMS and volume and rs-FC and; (2) associations between volume and rs-FC and interactions of the three aspects of PNMS (objective hardship × subjective distress, objective hardship × cognitive appraisal, subjective distress × cognitive appraisal). Significant interactions at the uncorrected level (*p* < 0.05) were probed with PROCESS macro (Hayes and Preacher, 2013) to identify regions of significance. The above regression analyses were FDR-corrected for multiple comparisons based on three aspects of PNMS and the number of brain regions in which volume and rs-FC showing between-group differences.

## 3. Results

### 3.1. Larger volumes of the amygdala, hippocampus, and prefrontal cortex

#### 3.1.1. Amygdala volume

**FIGURE 3:**
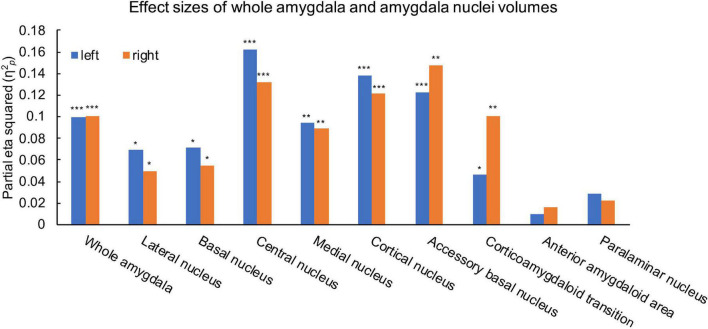
Effects size estimates measured by partial eta squared (η^2^_*p*_) for the left (blue) and right (orange) whole amygdala and amygdala nuclei. η^2^_*p*_ = 0.01 indicates a small effect; η^2^_*p*_ = 0.06 indicates a medium effect, and η^2^_*p*_ = 0.14 indicates a large effect. ***q < 0.001, **q < 0.01, *q < 0.05.

Ice Storm participants had significantly larger bilateral whole amygdala volumes compared to controls (left: *p* = 0.001; right: *p* = 0.001, Table 1). Ice Storm participants had larger volumes, that survived FDR correction, of bilateral lateral, basal, central, medial, cortical, accessory basal nuclei, and corticoamygdaloid transition compared to controls; left central and right accessory basal nuclei showed large effect sizes (Table 1 and Figure 3). The volumes of anterior amygdaloid area and paralaminar nucleus did not differ between the two groups (Table 1).

**TABLE 1:** Ice Storm participants had larger volumes of whole amygdala, amygdala nuclei, whole hippocampus, hippocampal subfields, and prefrontal subregions.

Regions	Hemisphere	Ice Storm (*n* = 39) mean ± SE^§^	Controls (*n* = 65) mean ± SE^§^	ANCOVA results^¶^ *F* score, *p* value, *q* value, partial eta squared (η^2^_*p*_)
**Whole amygdala**				
	Left	2286.15 ± 35.32	2118.55 ± 24.72	*F*_1_,_99_ = 11.04, *p* = **0.001**, η^2^_*p*_ = 0.100
	Right	2378.02 ± 35.07	2211.11 ± 24.55	*F*_1_,_99_ = 11.11, *p* = **0.001**, η^2^_*p*_ = 0.101
**Lateral nucleus**				
	Left	856.48 ± 14.25	801.33 ± 9.98	*F*_1_,_99_ = 7.35, *p* = 0.008, *q* = **0.013**, η^2^_*p*_ = 0.069
	Right	887.18 ± 15.92	835.50 ± 11.15	*F*_1_,_99_ = 5.17, *p* = 0.025, *q* = **0.035**, η^2^_*p*_ = 0.050
**Basal nucleus**				
	Left	562.09 ± 9.95	523.12 ± 6.96	*F*_1_,_99_ = 7.53, *p* = 0.007, *q* = **0.013**, η^2^_*p*_ = 0.071
	Right	581.96 ± 9.91	547.99 ± 6.94	*F*_1_,_99_ = 5.76, *p* = 0.018, *q* = **0.027**, η^2^_*p*_ = 0.055
**Central nucleus**				
	Left	70.34 ± 2.03	57.68 ± 1.42	*F*_1_,_99_ = 19.09, p = 0.000031, *q* = **0.0006**, η^2^_*p*_ = **0.162**
	Right	73.07 ± 2.17	61.04 ± 1.52	F_1_,_99_ = 15.04, p = 0.000189, q = **0.001**, η^2^_*p*_ = 0.132
**Medial nucleus**				
	Left	35.02 ± 1.49	28.20 ± 1.04	*F*_1_,_99_ = 10.28, *p* = 0.0018, *q* = **0.004**, η^2^_*p*_ = 0.094
	Right	36.03 ± 1.46	29.51 ± 1.02	*F*_1_,_99_ = 9.72, *p* = 0.0024, *q* = **0.005**, η^2^_*p*_ = 0.089
**Cortical nucleus**				
	Left	39.43 ± 0.90	34.32 ± 0.63	*F*_1_,_99_ = 15.80, *p* = 0.000134, *q* = **0.001**, η^2^_*p*_ = 0.138
	Right	41.33 ± 0.82	37.00 ± 0.58	*F*_1_,_99_ = 13.61, *p* = 0.000368, *q* = **0.001**, η^2^_*p*_ = 0.121
**Accessory basal nucleus**				
	Left	358.83 ± 5.94	327.20 ± 4.16	*F*_1_,_99_ = 13.92, *p* = 0.000319, *q* = **0.001**, η^2^_*p*_ = 0.123
	Right	377.55 ± 5.81	343.17 ± 4.07	*F*_1_,_99_ = 17.19, *p* = 0.00072, *q* = **0.002**, η^2^_*p*_ = **0.148**
**Corticoamygdaloid transition**				
	Left	233.72 ± 3.81	221.79 ± 2.67	*F*_1_,_99_ = 4.80, *p* = 0.031, *q* = **0.040**, η^2^_*p*_ = 0.046
	Right	243.03 ± 3.86	224.62 ± 2.70	*F*_1_,_99_ = 11.17, *p* = 0.001, *q* = **0.003**, η^2^_*p*_ = 0.101
**Anterior amygdaloid area**				
	Left	67.43 ± 1.61	65.11 ± 1.13	*F*_1_,_99_ = 1.02, *p* = 0.315, q = 0.315, η^2^_*p*_ = 0.010
	Right	74.26 ± 1.64	71.25 ± 1.15	*F*_1_,_99_ = 1.65, *p* = 0.202, *q* = 0.214, η^2^_*p*_ = 0.016
**Paralaminar nucleus**				
	Left	62.81 ± 1.22	59.81 ± 0.85	*F*_1_,_99_ = 2.97, *p* = 0.088, *q* = 0.106, η^2^_*p*_ = 0.029
	Right	63.62 ± 1.21	61.03 ± 0.85	*F*_1_,_99_ = 2.26, *p* = 0.136, *q* = 0.153, η^2^_*p*_ = 0.022
**Whole hippocampus**				
	Left	4606.83 ± 57.02	4378.66 ± 39.92	*F*_1_,_99_ = 7.85, p** = 0.006**, η^2^_*p*_ = 0.073
	Right	4714.22 ± 54.99	4484.03 ± 38.50	*F*_1_,_99_ = 8.59, *p* **= 0.004**, η^2^_*p*_ = 0.080
**Parasubiculum**				
	Left	86.33 ± 2.15	85.43 ± 1.50	*F*_1_,_99_ = 0.09, *p* = 0.770, *q* = 0.770, η^2^_*p*_ = 0.001
	Right	84.53 ± 2.50	82.59 ± 1.75	*F*_1_,_99_ = 0.30, *p* = 0.588, *q* = 0.627, η^2^_*p*_ = 0.003
**HATA**				
	Left	77.35 ± 1.81	72.20 ± 1.27	*F*_1_,_99_ = 3.98, *p* = 0.049, *q* = 0.078, η^2^_*p*_ = 0.039
	Right	83.25 ± 1.92	73.02 ± 1.35	*F*_1_,_99_ = 13.86, *p* = 0.000327, *q* = **0.004**, η^2^_*p*_ = 0.123
**Fimbria**				
	Left	104.51 ± 3.85	112.78 ± 2.70	*F*_1_,_99_ = 2.26, *p* = 0.136, *q* = 0.181, η^2^_*p*_ = 0.022
	Right	95.50 ± 3.99	108.88 ± 2.80	*F*_1_,_99_ = 5.49, *p* = 0.021, *q* = **0.046**, η^2^_*p*_ = 0.053
**Hippocampal fissure**				
	Left	212.95 ± 6.90	190.37 ± 4.83	*F*_1_,_99_ = 5.26, *p* = 0.024, *q* = **0.046**, η^2^_*p*_ = 0.050
	Right	200.05 ± 5.45	195.96 ± 3.82	*F*_1_,_99_ = 0.28, *p* = 0.601, *q* = 0.627, η^2^_*p*_ = 0.003
**HP tail**				
	Left	812.14 ± 14.91	750.73 ± 10.44	*F*_1_,_99_ = 8.32, *p* = 0.005, *q* = **0.015**, η^2^_*p*_ = 0.078
	Right	803.75 ± 14.94	748.48 ± 10.46	*F*_1_,_99_ = 6.71, *p* = 0.011, *q* = **0.029**, η^2^_*p*_ = 0.063
**Presubiculum**				
	Left	410.98 ± 7.02	420.12 ± 4.92	*F*_1_,_99_ = 0.83, *p* = 0.364, *q* = 0.421, η^2^_*p*_ = 0.008
	Right	380.34 ± 7.79	394.37 ± 5.45	*F*_1_,_99_ = 1.59, *p* = 0.210, *q* = 0.265, η^2^_*p*_ = 0.016
**Subiculum**				
	Left	568.95 ± 8.38	544.27 ± 5.87	*F*_1_,_99_ = 4.25, p = 0.042, *q* = 0.072, η^2^_*p*_ = 0.041
	Right	551.78 ± 7.13	542.57 ± 4.99	*F*_1_,_99_ = 0.82, *p* = 0.368, *q* = 0.421, η^2^_*p*_ = 0.008
**CA1**				
	Left	820.77 ± 15.27	781.98 ± 10.69	F_1_,_99_ = 3.16, *p* = 0.078, *q* = 0.110, η^2^_*p*_ = 0.031
	Right	883.15 ± 14.03	834.17 ± 9.83	*F*_1_,_99_ = 5.97, *p* = 0.016, *q* = **0.038**, η^2^_*p*_ = 0.057
**CA3**				
	Left	284.65 ± 7.19	252.59 ± 5.03	*F*_1_,_99_ = 9.76, *p* = 0.0023, *q* = **0.008**, η^2^_*p*_ = 0.090
	Right	321.75 ± 6.58	279.72 ± 4.60	F_1_,_99_ = 20.03, p = 0.00002, q = **0.0005**, η^2^_*p*_ = **0.168**
**CA4**				
	Left	328.90 ± 5.36	304.99 ± 3.75	*F*_1_,_99_ = 9.74, *p* = 0.0024, *q* = **0.008**, η^2^_*p*_ = 0.090
	Right	348.50 ± 5.50	321.21 ± 3.85	*F*_1_,_99_ = 12.06, *p* = 0.001, *q* = **0.005**, η^2^_*p*_ = 0.109
**GC ML DG**				
	Left	383.79 ± 5.79	356.39 ± 4.06	*F*_1_,_99_ = 10.97, *p* = 0.001, *q* = **0.005**, η^2^_*p*_ = 0.100
	Right	403.73 ± 6.22	374.37 ± 4.36	*F*_1_,_99_ = 10.92, *p* = 0.001, *q* = **0.005**, η^2^_*p*_ = 0.099
**Molecular layer HP**				
	Left	728.47 ± 11.29	697.18 ± 7.90	*F*_1_,_99_ = 3.77, *p* = 0.055, *q* = 0.083, η^2^_*p*_ = 0.037
	Right	757.94 ± 10.26	724.64 ± 7.19	*F*_1_,_99_ = 5.16, *p* = 0.025, *q* = **0.046**, η^2^_*p*_ = 0.050
**Prefrontal Caudal anterior cingulate**				
	Left	2799.34 ± 88.09	2798.81 ± 61.67	*F*_1_,_99_ < 0.01, *p* = 0.997, *q* = 0.997, η^2^_*p*_ < 0.001
	Right	3213.85 ± 60.64	3086.09 ± 42.45	*F*_1_,_99_ = 2.18, *p* = 0.143, *q* = 0.310, η^2^_*p*_ = 0.022
**Caudal middle frontal**				
	Left	9483.21 ± 232.58	9687.43 ± 162.82	F_1_,_99_ = 0.38, *p* = 0.540, *q* = 0.665, η^2^_*p*_ = 0.004
	Right	8805.24 ± 223.75	9289.21 ± 156.64	*F*_1_,_99_ = 2.29, *p* = 0.133, *q* = 0.310, η^2^_*p*_ = 0.023
**Lateral orbitofrontal**				
	Left	10618.47 ± 145.27	10488.23 ± 101.70	*F*_1_,_99_ = 0.39, *p* = 0.532, *q* = 0.665, η^2^_*p*_ = 0.004
	Right	10134.26 ± 174.67	10290.02 ± 122.28	*F*_1_,_99_ = 0.39, *p* = 0.534, *q* = 0.665, η^2^_*p*_ = 0.004
**Medial orbitofrontal**				
	Left	7007.03 ± 117.46	7313.12 ± 82.23	*F*_1_,_99_ = 3.33, *p* = 0.071, *q* = 0.310, η^2^_*p*_ = 0.033
	Right	7551.49 ± 119.60	7548.77 ± 83.73	*F*_1_,_99_ < 0.01, *p* = 0.987, *q* = 0.997, η^2^_*p*_ < 0.001
**Rostral anterior cingulate**				
	Left	3545.86 ± 96.48	3561.41 ± 67.54	*F*_1_,_99_ = 0.013, *p* = 0.910, *q* = 0.997, η^2^_*p*_ < 0.001
	Right	2805.30 ± 71.10	2657.84 ± 50.48	*F*_1_,_99_ = 2.05, *p* = 0.155, *q* = 0.310, η^2^_*p*_ = 0.020
**Rostral middle frontal**				
	Left	21711.81 ± 393.30	22592.39 ± 275.34	*F*_1_,_99_ = 2.46, *p* = 0.120, *q* = 0.310, η^2^_*p*_ = 0.024
	Right	22040.68 ± 368.87	22836.27 ± 258.24	*F*_1_,_99_ = 2.28, *p* = 0.134, *q* = 0.310, η^2^_*p*_ = 0.023
**Superior frontal**				
	Left	35113.91 ± 430.27	32959.84 ± 301.22	*F*_1_,_99_ = 12.29, *p* = 0.001, *q* = **0.016**, η^2^_*p*_ = 0.110
	Right	33352.44 ± 415.27	31884.69 ± 290.72	*F*_1_,_99_ = 6.13, *p* = 0.015, *q* = 0.120, η^2^_*p*_ = 0.058
**Frontal pole**				
	Left	1296.12 ± 53.53	1387.99 ± 37.48	*F*_1_,_99_ = 1.44, *p* = 0.232, *q* = 0.412, η^2^_*p*_ = 0.014
	Right	1544.56 ± 58.55	1638.51 ± 40.99	*F*_1_,_99_ = 1.26, *p* = 0.264, *q* = 0.422, η^2^_*p*_ = 0.013

ANCOVA, analysis of covariance; SE, standard error.

^§^Marginal mean and SE with controlling for T1w Repetition Time (TR), Echo Time (TE), and sex.

^¶^Between-group difference with controlling for T1w, TR, TE, and sex.

Bold *p* values indicate significant results for the whole amygdala and the whole hippocampus.

Bold *q* values indicate significant results at FDR-corrected threshold for 18 comparisons based on 9 amygdala nuclei (left and right), 24 comparisons based on 12 hippocampal subfields (left and right), 16 comparisons based on 8 prefrontal subregions (left and right).

Bold η^2^_*p*_ values indicate large effect size (>0.14).

#### 3.1.2. Hippocampal volume

**FIGURE 4:**
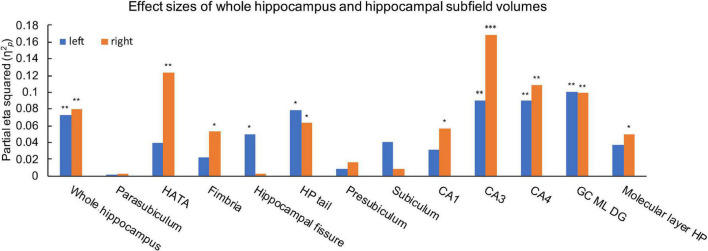
Effects size estimates measured by partial eta squared (η^2^_*p*_) for the left (blue) and right (orange) whole hippocampus and hippocampal subfields. η^2^_*p*_ = 0.01 indicates a small effect; η^2^_*p*_ = 0.06 indicates a medium effect, and η^2^_*p*_ = 0.14 indicates a large effect. ***q < 0.001, **q < 0.01, *q < 0.05.

Ice Storm participants had significantly larger bilateral whole hippocampal volumes compared to controls (left: *p* = 0.006; right: *p* = 0.004, Table 1). Ice Storm participants had larger volumes, that survived FDR correction, of the right CA1, the right HATA, the right molecular layer, the left fissure, bilateral tail, bilateral CA3, bilateral CA4, and bilateral DG, but smaller right fimbria volume compared to controls; right CA3 showed large effect size (Table 1 and Figure 4).

#### 3.1.3. Prefrontal cortex volume

Ice Storm participants had larger left superior frontal volume, that survived FDR correction, compared to controls (*p* = 0.001, *q* = 0.016), while other remaining prefrontal subregions did not show significant between-group volumetric difference (Table 1).

### 3.2. Associations between PNMS and volumes of amygdala, hippocampus, and prefrontal cortex within the ice storm group (none survived FDR correction)

#### 3.2.1. Amygdala volume

The linear regression results were FDR-corrected for 42 comparisons based on three aspects of PNMS and 14 amygdala nuclei showing between-group volumetric differences. No main effects of PNMS were observed for amygdala nuclei volumes. An interaction between objective hardship and subjective distress was observed for the right medial nucleus volume (*p* = 0.010): larger right medial nucleus volume was seen with a combination of higher objective hardship and higher subjective distress (Supplementary Figure 3). However, this interaction did not survive FDR correction (*q* = 0.420). An interaction of subjective distress and cognitive appraisal was observed for the right medial nucleus volume (*p* = 0.028): larger right medial nucleus volume was seen with a combination of higher subjective distress and more negative cognitive appraisal (Supplementary Figure 4). However, this interaction did not survive FDR correction (*q* = 0.588). An interaction of objective hardship and subjective distress was observed for the right cortical nucleus volume (*p* = 0.049): larger right cortical nucleus volume was seen with a combination of higher objective hardship and higher subjective distress (Supplementary Figure 5). However, this interaction did not survive FDR correction (*q* = 0.686).

#### 3.2.2. Hippocampal volume

The linear regression results were FDR-corrected for 39 comparisons based on three aspects of PNMS and 13 hippocampal subfields showing between-group volumetric differences. The lower the maternal subjective distress, the larger the young adult offspring’s right hippocampal tail volume (beta = −0.437, *p* = 0.006, Supplementary Figure 6). However, this main effect did not survive FDR correction (*q* = 0.234). Similarly, we observed an interaction between subjective distress and cognitive appraisal for the left hippocampal tail volume (*p* = 0.046): larger left hippocampal tail volume was seen with a combination of lower subjective distress and more positive cognitive appraisal (Supplementary Figure 7). However, this interaction did not survive FDR correction (*q* = 0.322). In addition, we observed an interaction between objective hardship and subjective distress on the right CA4 volume (*p* = 0.049): larger right CA4 volume was seen with a combination of higher objective hardship and higher subjective distress (Supplementary Figure 8). However, this interaction did not survive FDR correction (*q* = 0.322). We also observed an interaction of subjective distress and cognitive appraisal on the right CA4 volume (*p* = 0.034). When we probed this interaction, there were no regions of significance (Supplementary Figure 9), and this interaction did not survive FDR correction (*q* = 0.322). Likewise, we found an interaction between objective hardship and subjective distress on the right DG volume (*p* = 0.041): larger right DG volume was seen with a combination of higher objective hardship and higher subjective distress (Supplementary Figure 10). However, this interaction did not survive FDR correction (q = 0.322). We also observed an interaction between subjective distress and cognitive appraisal on the right DG volume (*p* = 0.049), but there were no regions of significance (Supplementary Figure 11), and this interaction did not survive FDR correction (*q* = 0.322).

#### 3.2.3. Prefrontal cortex volume

No main effects nor interactions were observed between PNMS and the left superior frontal volume.

### 3.3. Lower amygdala-prefrontal rs-FC

Compared to controls, Ice Storm participants exhibited lower rs-FC between the right whole amygdala and (1) the left caudal middle frontal (*p* = 0.003, *q* = 0.015); (2) the left superior frontal (*p* < 0.001, *q* = 0.003); (3) the left frontal pole (*p* < 0.001, *q* < 0.001), and (4) the right frontal pole (*p* = 0.005, *q* = 0.021) (Table 2). For the right amygdala nuclei, Ice Storm participants had lower rs-FC (1) from the right lateral nucleus to the left frontal pole; (2) from the right basal nucleus to the left superior frontal and the left caudal middle frontal; (3) from the right accessory basal nucleus to the right frontal pole and the left superior frontal; and (4) from the right paralaminar nucleus to bilateral superior frontal and bilateral caudal middle frontal (Supplementary Table 4 and Supplementary Figure 12).

**TABLE 2:** Ice Storm participants had lower amygdala-prefrontal rs-FC and lower hippocampus-prefrontal rs-FC compared to controls.

Regions	Beta	*T* score	*p*	*q*
**Seed: Right whole amygdala**				
Left frontal pole	−0.18	−3.69	0.000368	**0.0003**
Left superior frontal	−0.21	−3.65	0.000421	**0.0034**
Left caudal middle frontal	−0.18	−3.07	0.002783	**0.0148**
Right frontal pole	−0.14	−2.86	0.005190	**0.0208**
**Seed: Right hippocampal tail**				
Left lateral orbitofrontal	−0.25	−4.66	0.000010	**0.0002**

rs-FC, resting-state functional connectivity.

Bold *q* values indicate significant results at FDR-corrected threshold.

### 3.4. Lower hippocampus-prefrontal rs-FC

Ice Storm participants exhibited significantly lower FDR-corrected rs-FC between the right hippocampal tail and the left lateral orbitofrontal than controls (*p* < 0.001, *q* < 0.001) (Table 2). In addition, Ice Storm participants had lower rs-FC from the left parasubiculum to bilateral superior frontal and the right frontal pole compared to controls (shown in Supplementary Table 4 and Supplementary Figure 12).

### 3.5. Associations between PNMS and whole amygdala-prefrontal rs-FC within the Ice Storm group (none survived FDR correction)

No main effects of PNMS were observed for whole amygdala-prefrontal rs-FC. An interaction between objective hardship and cognitive appraisal was observed for rs-FC between the right whole amygdala and the right frontal pole (*p* = 0.022): lower rs-FC between the right whole amygdala and the right frontal pole was seen with a combination of higher objective hardship and more negative cognitive appraisal (Supplementary Figure 13). However, this interaction did not survive FDR correction (*q* = 0.264) for 12 comparisons based on three aspects of PNMS and four whole amygdala-prefrontal rs-FC showing between-group differences.

### 3.6. Associations between PNMS and hippocampal tail-prefrontal rs-FC within the ice storm group (none survived FDR correction)

The higher the maternal objective hardship, the lower rs-FC between the right hippocampal tail and the left lateral orbitofrontal (beta = −0.361, *p* = 0.028; Supplementary Figure 14). This main effect did not survive FDR correction (*q* = 0.084) for three comparisons based on three aspects of PNMS.

## 4. Discussion

To our knowledge, this is the first study examining volumes and rs-FC of the amygdala, hippocampus and prefrontal cortex in young adult offspring exposed *in utero* to varying levels of disaster-related PNMS. Primarily, we found that, compared to controls, young adult offspring exposed to disaster-related PNMS had larger volumes of the amygdala, hippocampus, and prefrontal cortex but lower amygdala-prefrontal connectivity and lower hippocampus-prefrontal connectivity. In addition, within the Ice Storm group, we found several associations between the severity of objective hardship or subjective distress, or interactions between PNMS variables, that explained variance in volume or functional connectivity, although none survived FDR corrections.

We observed that Ice Storm participants had larger bilateral whole amygdala volumes than controls. Larger amygdala may not be adaptive given that our previous Project Ice Storm findings at age 11½ indicated that larger right whole amygdala volume was associated with more severe externalizing behaviors (Jones et al., 2019). Our current results are inconsistent with a previous study finding that pre-natal stressful life events (e.g., break-up or divorce from partner, consideration of abortion, violence, serious illness or death in the family, financial difficulties) were associated with decreased whole amygdala volume in young adulthood (Mareckova et al., 2021). This discrepancy from our finding might be mainly attributed to different operationalizations of stress exposure; most of the stressors in the previous study (Mareckova et al., 2021) could have been brought on by parental characteristics that increase propensity to create stressful life events and that also confer genetic risk to the offspring that might be seen in amygdala development, whereas exposure to a natural disaster is an “independent” stressor that is outside of the control of the individual. In that sense, Project Ice Storm could be considered a natural experiment, and the current comparison between Ice Storm-exposed participants and controls involves random assignment to groups. Another possible explanation is that the stressors captured in the previous study (Mareckova et al., 2021) were all from the first half of pregnancy while Project Ice Storm included exposures from 3-months preconception to the very end of pregnancy. Although our previous Ice Storm results at age 11½ found that larger right whole amygdala volume was associated higher maternal subjective distress during 2^nd^ and 3^rd^ trimesters of pregnancy in boys, and higher maternal objective hardship in girls (Jones et al., 2019), at age 19 we found no associations between the severity of PNMS and bilateral whole amygdala volumes. It is possible, then, that the mere exposure to the ice storm *in utero* is the active ingredient in the effect on whole amygdala volume that persists into young adulthood.

Regarding amygdala nuclei, we found that, compared to controls, Ice Storm participants exhibited larger volumes of bilateral lateral, basal, central, medial, cortical, accessory basal nuclei, and corticoamygdaloid transition, but not of the anterior amygdaloid area or paralaminar nucleus. The absence of volumetric increases in the latter nuclei might be, in part, due to their low density of binding receptors for stress-inducing hormones and neurotransmitters (LeDoux, 2007). Evidence from rodents showed that glucocorticoids can directly bind to glucocorticoid receptors in the basolateral complex, consisting of lateral, basal, accessory basal and paralaminar nuclei, in which brief stress exposure triggers an increase in the spine density (Roozendaal et al., 2009). The basolateral complex is the largest and is the main input site of the amygdala (deCampo and Fudge, 2012). Central and medial nuclei, the main output area, are involved in processing glucocorticoid signaling and regulating autonomic, behavioral and hormonal response to stress *via* efferent projections to hypothalamus and the bed nucleus of the stria terminals. The superficial complex, including cortical, corticoamygdaloid transition and anterior amygdaloid area, are the major targets of olfactory projections (Sah et al., 2003), and are involved in selective social processing of the sensory inputs (Goossens et al., 2009). The volumetric increases in these nuclei may reflect stress-sensitive neurodevelopment *in utero*.

Unlike larger amygdala volume, Ice Storm participants exhibited lower rs-FC between the prefrontal cortex and the right whole amygdala, driven by the right basolateral complex. It is evident that basal and lateral nuclei receive fearful inputs from the visual cortex and project to prefrontal cortex (Ressler, 2010). Upon acute stress, the amygdala overactivates while the medial prefrontal cortex deactivates (Herman et al., 2005). The medial prefrontal cortex is largely inhibitory to HPA axis secretion, which in turn deactivates the amygdala (Bremner, 2006; Dedovic et al., 2009). Our finding suggests inadequate integration of amygdala fear acquisition into the prefrontal processing circuit in response to disaster-related PNMS.

As for the hippocampus, we found that, compared to controls, Ice Storm participants had larger bilateral whole hippocampal volumes. In typically developing general populations, hippocampal volume has been reported to increase with age from childhood to young adulthood (Xu et al., 2020). Our results of larger bilateral whole hippocampal volumes may be explained, in part, by the “predictive adaptive response hypothesis” (Gluckman et al., 2005), which indicates that exposure to stress in the intra-utero environment biases protective stress responses to better adapt the development of an organism to the ex-utero environment (Sandman et al., 2012; Glover et al., 2017) by overdevelopment of the hippocampus. Further, we identified hippocampal subfield-specific volumetric alterations: larger volumes of the HATA, CA1, molecular layer, fissure, tail, CA3, CA4, and DG, but smaller fimbria volume. The HATA is tightly co-located and interconnected with the amygdala at the cellular level, and is involved in memory processing (Li et al., 2021); the CA1 is active in pattern completion while the CA3 is active in pattern separation, and the DG is involved in both processes (Postel et al., 2019). Among the hippocampal subfields, the vulnerability of the CA3 has been most-frequently reported possibly due to its role as the main target for glucocorticoids (Cai et al., 2007; Jia et al., 2010; Marečková et al., 2018; Zhang et al., 2019). In line with this, the CA3 in our study was the most vulnerable with the largest between-group effect size (η^2^_*p*_ = 0.168). In contrast, the smaller right fimbria volume possibly indicates inconsistent regulatory roles of the hippocampal subfields in response to PNMS. The hippocampal tail is relevant for spatial information and negative emotion (Li et al., 2021). Our study extends prior research showing larger right hippocampal tail volume, but lower rs-FC between the right hippocampal tail and the left lateral orbitofrontal in young adult offspring of mothers exposed to natural disaster-related pre-natal stress. Interestingly, the whole hippocampus rs-FC did not differ between Ice Storm participants and controls, suggesting that subfield rs-FC, instead of whole hippocampus rs-FC, better underlies the neuropathology of PNMS.

In our study, Ice Storm participants had larger left superior frontal volume than controls. The superior frontal, located at the superior part of the prefrontal cortex, has been reported to be involved in motor control tasks, working memory, and higher cognitive processing (Li et al., 2013). We found that, compared to controls, Ice Storm participants had lower rs-FC between the right whole amygdala and the left superior frontal extending to the left caudal middle frontal and bilateral frontal poles, and lower rs-FC between the right hippocampal tail and the left lateral orbitofrontal cortex, which consistently point to decreased prefrontal integration in amygdala and hippocampal circuitry of pre-natal stress. Further, these results suggest that the prefrontal integration of the amygdala and hippocampal information is subregion-specific. It has been reported that the left superior frontal gyrus mainly contributes to working memory (du Boisgueheneuc et al., 2006) and the left caudal middle frontal gyrus mainly contributes to self-initiated elaborative strategies (Husa et al., 2017); frontal poles are mainly involved in action selection (Kovach et al., 2012). We propose that these subregions contribute differently to the processes of high-level cognitive functions *via* integrating information from the amygdala. Lateral orbitofrontal cortex mainly contributes to decision-making by combining prior with current information (Nogueira et al., 2017). In addition, the connectivity between lateral orbitofrontal cortex and hippocampal tail may process memory consolidation of information.

Within the Ice Storm group, we tested associations between the severity of PNMS and amygdala nuclei volumes at the uncorrected level. These overall trends extend our previous findings of PNMS influences on whole amygdala volume at age 11½ (Jones et al., 2019) to specific amygdala nuclei at age 19. We found that higher objective hardship, higher subjective distress and more negative cognitive appraisal interacted to predict larger medial nucleus volume, and that higher objective hardship and higher subjective distress interacted to predict larger volumes of medial and cortical nuclei. However, these findings should be interpreted with caution given that none of these interactions survived FDR correction.

Similar to the whole amygdala, no robustly significant associations were observed between the severity of PNMS and the whole hippocampal volume, in line with previous reports of no associations between pre-natal stressful life events and the whole hippocampal volume in young adult offspring (Favaro et al., 2015; Marečková et al., 2018). We also tested the associations between the severity of PNMS and hippocampal subfield volumes; none of the results survived FDR correction. The results from Project Ice Storm at age 11½ indicated that (1) greater maternal objective hardship was associated with increased right whole hippocampal volume in girls; (2) COMT genotype moderated the association between maternal objective hardship and right whole hippocampal volume in boys; and (3) COMT genotype moderated the association between maternal subjective distress and right whole hippocampal volume in girls (Cao-Lei et al., 2021). Some differences between age 11½ and the current study are that the age 11½ analyses were conducted with whole hippocampal volume only, and that the age 11½ analyses were conducted with boys and girls separately. In comparison, the current study further included analyses at the level of hippocampal subfields. In this sense, future studies may be needed to identify whether genetics and sex moderate associations between PNMS and hippocampal subfield volumes.

Within the Ice Storm group, we tested associations between the severity of PNMS and hippocampal subfield volumes at the uncorrected level. These results are worth mentioning, despite their failure to survive FDR corrections, in order to inform future hypotheses. Unlike our findings suggesting that higher PNMS is consistently associated with larger amygdala nuclei, these findings suggest that PNMS predicts larger hippocampal subfield volumes in a stress- and subfield- specific manner (Herman et al., 2005): higher subjective distress predicted smaller tail volume while higher objective hardship and higher subjective distress interacted to predict larger volumes of CA4 and DG. We speculate that the observed inconsistency might be attributed to much higher heterogeneity for hippocampal subfields than for amygdala nuclei. Within the Ice Storm group, at the uncorrected level, we observed that a combination of higher maternal objective hardship and more negative maternal cognitive appraisal jointly predicted lower offspring amygdala-frontal rs-FC whereas higher maternal objective hardship predicted lower offspring hippocampus-lateral orbitofrontal rs-FC, indicating that prefrontal regulatory circuits of amygdala and hippocampus might be stress- and subregion- specific. As noted, however, these findings should be interpreted with caution given that the aforementioned main effects and interactions failed to survive FDR correction.

Despite the observed group differences, and the ability of the PNMS to predict amygdala and hippocampal structure and function within the Ice Storm group, one must be cautious in making causal interpretations. It is possible that the results reflect the direct effects of *in utero* exposure to PNMS on the fetal brain that endures to age 19 years. It may also be the case, however, that the PNMS created a physiological vulnerability to post-natal environmental stressors that could be responsible for indirect effects on brain 19 years later.

There were some limitations. First, our sample was small which limited the ability to go deeper in our analyses to test moderations by sex and timing *in utero*. The sample size also limits our ability to determine the extent to which reactivity to any post-natal environmental stressors might have mediated associations between PNMS and offspring brain at age 19. Second, since MRI data for Ice Storm and control groups were collected from different scanning sites, we minimized this limitation by controlling for the key scanning parameters. Third, the socioeconomic status of the families in this study is higher than the median of the population from which they were recruited in 1998, such that the current results may not generalize to lower class populations.

In conclusion, our findings highlight long-lasting effects of maternal stress before birth on the volumes and rs-FC of the amygdala, the hippocampus and the prefrontal cortex 19 years after exposure, in which their vulnerability might be stress- and subdivision- specific.

## Data availability statement

The raw data supporting the conclusions of this article will be made available by the authors, without undue reservation.

## Ethics statement

All phases of Project Ice Storm were approved by the Research Ethics Board of Douglas Mental Health University Institute. All the participants provided written informed consent.

## Author contributions

XL contributed to the design, control MRI data acquisition, all analyses, and the draft of the manuscript. MQ contributed to the volume and rs-FC analysis methodology. DL contributed to the statistical analysis and MRI data acquisition. GE contributed to the statistical analyses. SJ contributed to the Ice Storm MRI data acquisition. SK and PR-N contributed to the supervision. All authors have approved the final manuscript.

## Footnotes

1
https://fcon_1000.projects.nitrc.org/indi/abide/

2
https://fcon_1000.projects.nitrc.org/indi/adhd200/
