# Supplementary material for: Neural correlates of disaster-related prenatal maternal stress in young adults from Project Ice Storm: Focus on amygdala, hippocampus, and prefrontal cortex
Source: Front Hum Neurosci. 2023 Feb 1;17:1094039. doi: 10.3389/fnhum.2023.1094039 (PMC9929467; doi:10.3389/fnhum.2023.1094039)
Supplement: Supplementary file 1 [file Data_Sheet_1.docx]

**Table S1.** Comparison of the current 19-year-old sample with MRI scanning with the 1998 recruitment sample.

| Variables | Non-MRI scanning (n=137)  Mean (SD) | MRI scanning (n=39)  Mean (SD) | p value |
| --- | --- | --- | --- |
| Objective hardship | 10.74 (4.72) | 11.15 (4.48) | 0.624 |
| Subjective distress (log-transformed) | 2.05 (1.14) | 1.99 (1.09) | 0.771 |
| Cognitive appraisal | 0.60 (0.49) | 0.72 (0.46) | 0.184 |
| Timing of exposure (days) | 108.95 (96.53) | 74.74 (99.31) | 0.054 |
| SES | 30.60 (13.04) | 25.68 (10.40) | **0.032^*^** |

SD, standard deviation; SES, socioeconomic status.

Lower SES scores correspond to higher SES

Cognitive appraisal is defined as two-level options: “negative” and “positive” (combine “neutral” and “positive”).

**Table S2.** Characteristics of Ice Storm participants and controls.

|  | Volume | | |  |  | rs-FC | | |
| --- | --- | --- | --- | --- | --- | --- | --- | --- |
|  | Ice Storm (n=39) | Controls (n=65) | p value |  |  | Ice Storm (n=38) | Controls (n=65) | p value |
| Mothers |  |  |  |  |  |  |  |  |
| Objective hardship, mean±SD (range) | 11.15±4.48 (4-24) | n/a | -- |  |  | 11.32±4.42 (4-24) | n/a | -- |
| Subjective distress, mean±SD (range) | 11.02±10.96 (0-40) | n/a | -- |  |  | 11.21±11.05 (0-40) | n/a | -- |
| Cognitive appraisal |  |  |  |  |  |  |  |  |
| Negative cognitive appraisal, n (%) | 11 (28.2) | n/a | -- |  |  | 11 (28.9) | n/a | -- |
| Neutral cognitive appraisal, n (%) | 10 (25.6) | n/a | -- |  |  | 9 (23.7) | n/a | -- |
| Positive cognitive appraisal, n (%) | 18 (46.2) | n/a | -- |  |  | 18 (47.4) | n/a | -- |
| Timing of exposure |  |  |  |  |  |  |  |  |
| 3 months preconception, n (%) | 11 (28.2) | n/a | -- |  |  | 10 (26.3) | n/a | -- |
| 1st trimester of pregnancy, n (%) | 12 (30.8) | n/a | -- |  |  | 12 (31.6) | n/a | -- |
| 2nd trimester of pregnancy, n (%) | 9 (23.1) | n/a | -- |  |  | 9 (23.7) | n/a | -- |
| 3rd trimester of pregnancy, n (%) | 7 (17.9) | n/a | -- |  |  | 7 (18.4) | n/a | -- |
| Socioeconomic status |  |  |  |  |  |  |  |  |
| Middle class, % | 12 (30.8) | n/a | -- |  |  | 12 (31.6) | n/a | -- |
| Upper middle class, % | 18 (46.2) | n/a | -- |  |  | 17 (44.7) | n/a | -- |
| Upper class, % | 9 (23.1) | n/a | -- |  |  | 9 (23.7) | n/a | -- |
| Young adult offspring |  |  |  |  |  |  |  |  |
| Sex, F/M (F %) | 21/18 (53.8) | 35/30 (53.8) | 1.000 |  |  | 21/17 (55.3) | 35/30 (53.8) | 0.889 |
| Gestational age at birth (wk), mean±SD (range) | 39.57±1.26(33.43-41) | n/a | -- |  |  | 39.58±1.27  (33.43-41) | n/a | -- |
| Age at scan (yr), mean±SD (range) | 18.68±0.37 (18.10-19.62) | 18.67±0.73 (17.20-20.08) | 0.935 |  |  | 18.69±0.37 (18.10-19.62) | 18.67±0.73 (17.20-20.08) | 0.857 |
| Full-scale IQ, mean±SD (range) | 113.14±10.98 (90-137) | 113.20±11.15 (89-133) ^¶^ | 0.979 |  |  | 112.77±10.89 (90-137) | 113.20±11.15 (89-133) ^a^ | 0.855 |
| Handedness, R/L | 36/3 | 58/6 | 0.536 |  |  | 35/3 | 58/6 | 0.552 |
| Mean FD (in-scanner head motion), mean±SD (range) | n/a | n/a | -- |  |  | 0.11±0.06 (0.04-0.24) | 0.12±0.05 (0.04-0.36) | 0.472 |
| In-scanner eye status, open/closed | n/a | n/a | -- |  |  | 38/0 | 46/19 | <0.001 |

IQ, intelligence quotient; SD, standard deviation; F, female; M, male; FD, framewise displacement; rs-FC, resting-state functional connectivity.

^¶^ n=55, there are 10 missing values, but the IQs of the 10 participants are ≥ 80.

**Table S3.** Characteristics of controls obtained from nine different scanning sites.

|  | ABIDEI-UM | ABIDEI- CALTECH | ABIDEI-NYU | ABIDEI-Olin | ABIDEI-CMU | ABIDEI-USM | ABIDEII-IP | ADHD200- Pittsburgh | ADHD 200- NeuroImage |
| --- | --- | --- | --- | --- | --- | --- | --- | --- | --- |
| Variables | Mean±SD  [range] | Mean±SD  [range] | Mean±SD  [range] | Mean±SD  [range] | Mean±SD  [range] | Mean±SD  [range] | Mean±SD  [range] | Mean±SD  [range] | Mean±SD  [range] |
| n | 12 | 3 | 5 | 1 | 1 | 7 | 5 | 21 | 10 |
| Age | 18.18±0.63  [17.2-19.2] | 19.47±0.68 [18.7-20] | 17.95±0.91 [17.31-19.73] | 20 | 20 | 18.52±0.59  [18.14-19.76] | 18.98±0.85 [17.56-19.85] | 18.55±0.45 [17.84-19.66] | 18.92±0.87 [17.50-20.08] |
| Sex (F/M) | 5/7 | 1/2 | 1/4 | 1/0 | 1/0 | 0/7 | 5/0 | 15/6 | 6/4 |
| Full-scale IQ | 109.06±9.79 [92.5-125.5] | 114.67±3.21 [111-117] | 105±9.90  [98-129] | 127 | 124 | 110.14±15.82  [89-131] | 110.8±9.31  [100-125] | 113.90±11.55 [92-133] | --^¶^ |
| Handedness (R/L) | 10/2 | 3/0 | 5/0 | 1/0 | 1/0 | 7/0 | 3/2 | 20/1 | 9/1 |
| Eye status | open | closed | open | open | closed | open | closed | open | closed |
| Mean FD | 0.11±0.08 [0.06-0.36] | 0.11±0.01 [0.09-0.11] | 0.08±0.05 [0.04-0.12] | 0.18 | 0.17 | 0.12±0.04 [0.08-0.18] | 0.09±0.04 [0.06-0.15] | 0.13±0.05 [0.07-0.27] | 0.11±0.04 [0.07-0.19] |
| Scanner | 3 Tesla GE Signa | 3 Tesla SIEMENS TrioTim | 3 Tesla SIEMENS Allegra | 3 Tesla SIEMENS Allegra | 3 Tesla SIEMENS Verio | 3 Tesla SIEMENS TrioTim | 1.5 Tesla PHILIPS Intera Achieva | 3 Tesla SIEMENS Allegra | 3 Tesla SIEMENS Avanto |
| T1w parameters | MPRAGE sequence, 40 slices, TR=250ms, TE=5.7ms, slice thickness=3mm, Flip Angle=90°, matrix=256×256 | MPRAGE sequence, 176 slices, TR=1590ms,TE=2.73ms, slice thickness=1mm, Flip Angle=10°,matrix=256×256 | MPRAGE sequence, 128 slices, TR=2530ms, TE=3.25ms, slice thickness=1.33mm, Flip Angle=7°, matrix=256×256 | MPRAGE sequence, 176 slices, TR=2500ms,TE=2.74ms, slice thickness=1mm, Flip Angle=8°, matrix=256×256 | MPRAGE sequence, 176 slices, TR=1870ms,TE=2.48ms, slice thickness=1mm, Flip Angle=8°, matrix=256×256 | MPRAGE sequence, 160 slices, TR=2300ms, TE=2.91ms, slice thickness=1.2mm, Flip Angle=9°, matrix=256×256 | MPRAGE sequence, 170 slices, TR=25ms, TE=5.6ms, slice thickness=1mm, Flip Angle=30°,matrix=256×256 | MPRAGE sequence, 176 slices, TR=2100ms,TE=3.43ms, slice thickness=1mm, Flip Angle=8°,matrix=256×256 | MPRAGE sequence, 176 slices, TR=2730ms, TE=2.95ms, slice thickness=1mm, Flip Angle=7°,matrix=256×256 |
| rs-fMRI parameters | EPI sequence, 40 slices, TR=2000ms, TE=30ms, Flip Angle=90°, slice thickness=3mm | EPI sequence, 34 slices, TR=2000ms,TE=30ms, Flip Angle=75°, slice thickness=3.5mm,FoV=224mm,matrix=64×64 | EPI sequence, 33 slices, TR=2000ms, TE=15ms, Flip Angle=90°, slice thickness=4mm, FoV=240mm, matrix=80×80 | EPI sequence, 29 slices, TR=1500ms, TE=27ms, Flip Angle=60°, slice thickness=4mm,FoV=220mm, matrix=64×64 | EPI sequence, 28 slices, TR=2000ms,TE=30ms, Flip Angle=73°,slice thickness=3mm,FoV=192mm,matrix=64×64 | EPI sequence, 40 slices, TR=2000ms, TE=28ms, Flip Angle=90°,slice thickness=3mm,FoV=220mm,matrix=64×64 | EPI sequence, 32 slices, TR=2700ms, TE=45ms, Flip Angle=90°,slice thickness=3.59mm,FoV=230mm,matrix=64×64 | EPI sequence, 29 slices, TR=1500ms, TE=29ms, Flip Angle=70°, slice thickness=4mm,FoV=200mm,matrix=64×64 | EPI sequence, 37 slices, TR=1960ms, TE=40ms, Flip Angle=80°, slice thickness=3mm,FoV=224mm,matrix=64×64 |

F, female; FD, framewise displacement; IQ, intelligence quotient; EPI, echo-planar imaging; M, male; MPRAGE, magnetization-prepared rapid gradient echo; L, left; R, right; SD, standard deviation; T1w, T1-weighted; rs-fMRI, resting-state functional magnetic resonance imaging; TR, repetition time; TE, echo time.

--^¶^ There are 10 missing values, but the IQs of the 10 participants are ≥ 80.


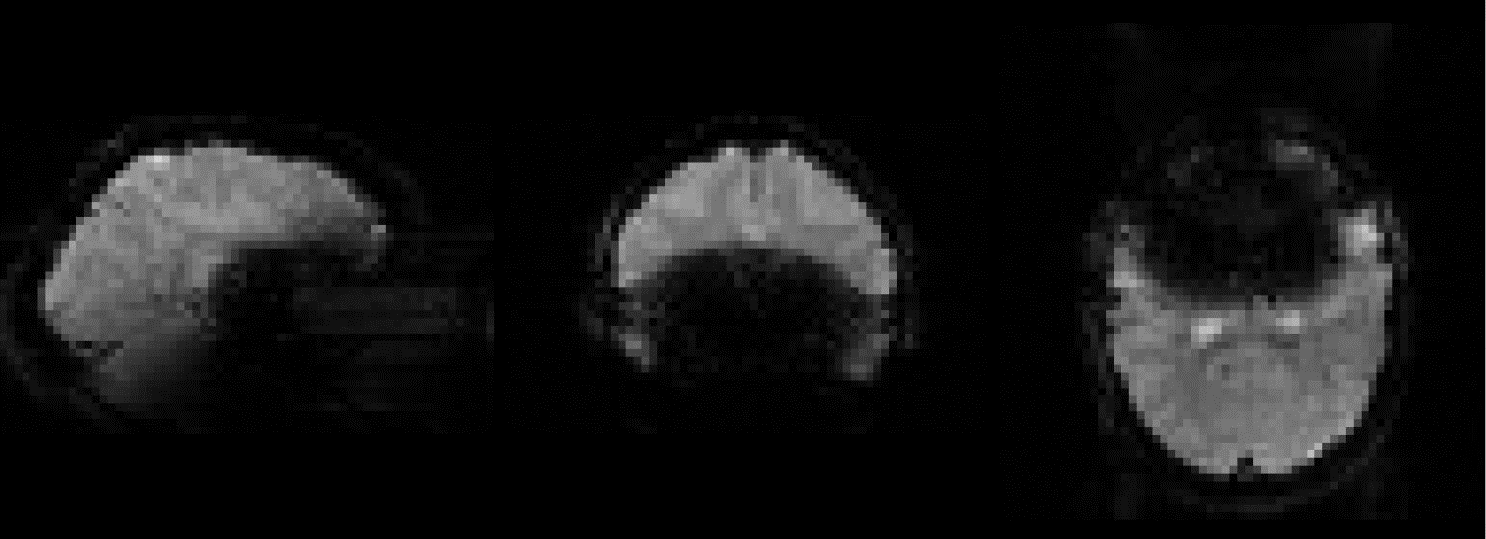


Sagittal

Coronal

Axial

**Figure S1.** One Ice Storm participant was excluded from rs-FC analyses due to rs-fMRI scan artifacts.

**
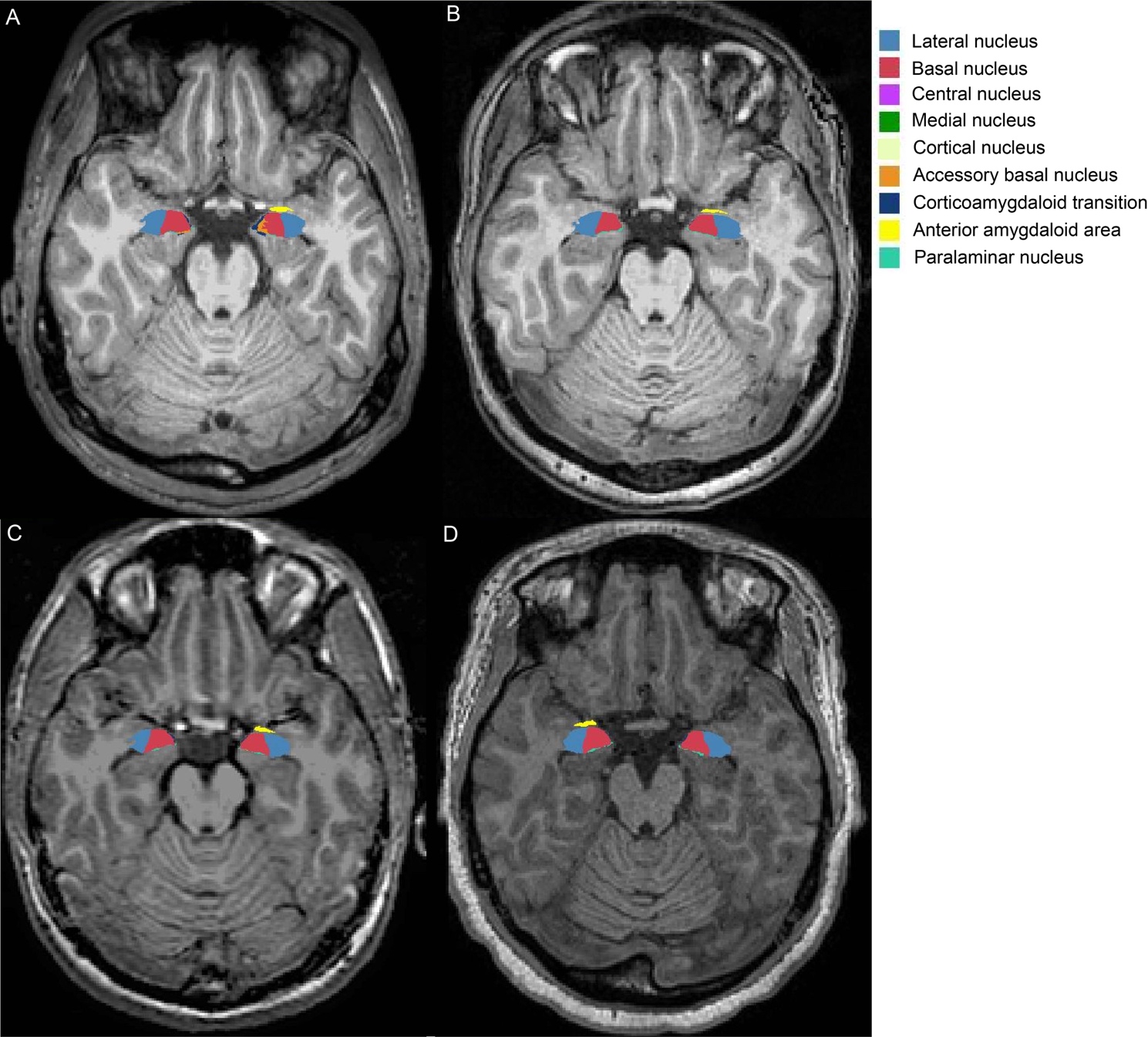
Figure S2**. Segmentation failure of the anterior amygdaloid area for four participants in the axial view. **A** Right anterior amygdaloid area was absent for one Ice Storm participant. **B-C** Right anterior amygdaloid area was absent for two controls. **D** Left anterior amygdaloid area was absent for one control.

**
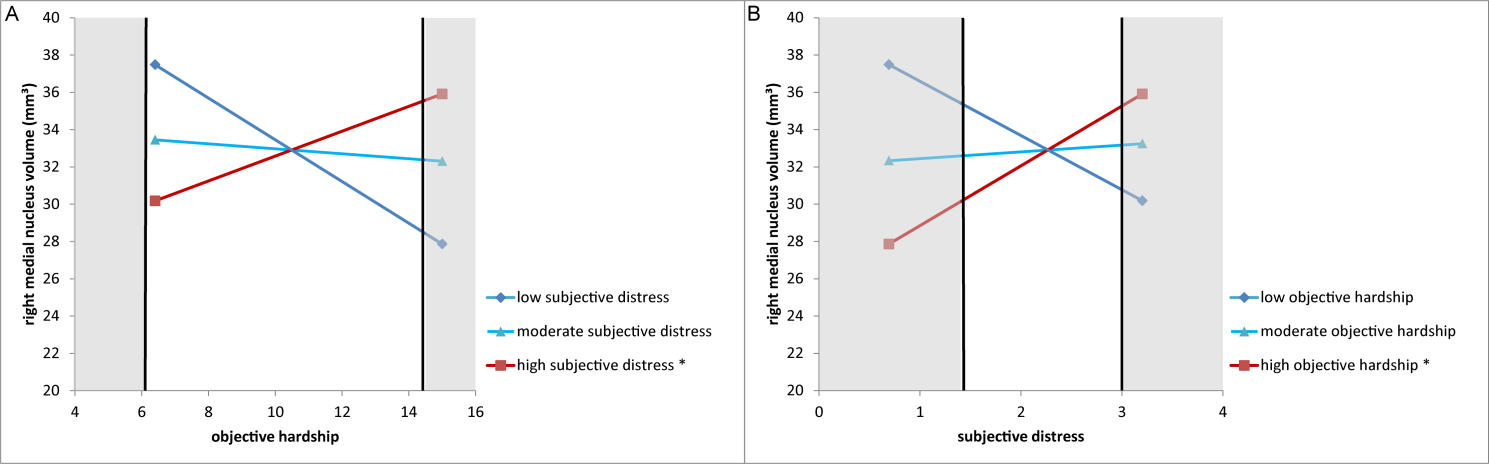
**

**Figure S3**. Interaction effects (p = 0.010, q = 0.420) between maternal objective hardship and subjective distress on the right medial nucleus volume in young adult offspring, controlling for sex. A. When maternal objective hardship was equal to or less than 6.10, lower maternal subjective distress was associated with larger right medial nucleus volume in the young adult offspring; when maternal objective hardship was equal to or larger than 14.42, higher maternal subjective distress was associated with larger right medial nucleus volume in young adult offspring. B. When maternal subjective distress was equal to or less than a log-transformed value of 0.68 (original scale value of 0.97), lower maternal objective hardship was associated with larger right medial nucleus volume in young adult offspring; when maternal subjective distress was equal to or larger than a log-transformed value of 3.00 (original scale value of 19.09), higher maternal objective hardship was associated with larger right medial nucleus volume in young adult offspring. The dark blue line represents low maternal subjective distress or low maternal objective hardship; the bright blue line represents moderate maternal subjective distress or moderate maternal objective hardship, and the red line represents high maternal subjective distress or high maternal objective hardship. Low, moderate and high maternal subjective distress lines are represented at the 16th, 50th and 84th sample percentiles of maternal subjective distress scores, respectively, which were at a log-transformed score of 0.69, 2.08 and 3.20 (original score of 0.99, 7.00 and 23.53), respectively. Low, moderate and high maternal objective hardship lines are represented at the 16th, 50th and 84th sample percentiles of maternal objective hardship scores, respectively, which were at score of 6.40, 11.00 and 15.00, respectively. The regions of significance (p < 0.05) are represented by the vertical line and the gray shading. ^*^ p < 0.05.

**
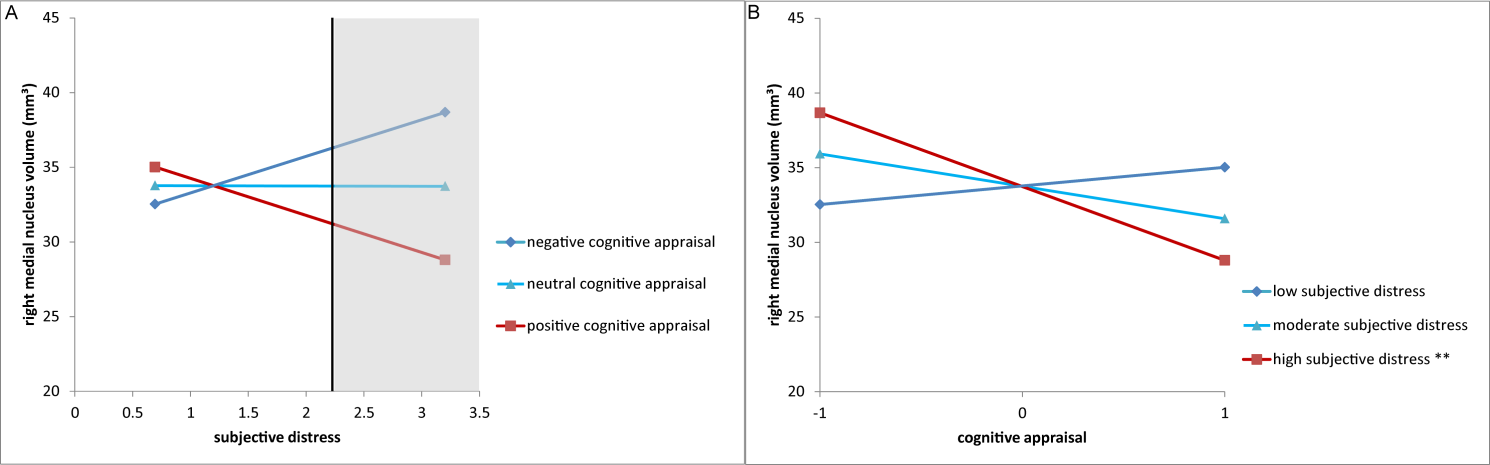
Figure S4**. Interaction effects (p = 0.028, q = 0.588) between maternal subjective distress and cognitive appraisal on the right medial nucleus volume in young adult offspring, controlling for sex. A: When maternal subjective distress was equal to or larger than a log-transformed value of 2.23 (original scale value of 8.30), maternal negative cognitive appraisal was associated with larger right medial nucleus volume in young adult offspring. The dark blue line represents maternal negative cognitive appraisal; the bright blue line represents maternal neutral cognitive appraisal, and the red line represents maternal positive cognitive appraisal. Negative, neutral and positive cognitive appraisal lines are represented at the 16th, 50th and 84th sample percentiles of maternal cognitive appraisal scores, respectively, which were at score of -1, 0 and 1, respectively. The region of significance (p < 0.05) is represented by the vertical line and the gray shading. B. The dark blue line represents low maternal subjective distress; the bright blue line represents moderate maternal subjective distress, and the red line represents high maternal subjective distress. Low, moderate and high maternal subjective distress lines are represented at the 16th, 50th and 84th sample percentiles of maternal subjective distress scores, respectively, which were at a log-transformed score of 0.69, 2.08 and 3.20 (original score of 0.99, 7.00 and 23.53), respectively. ^**^ p < 0.01

**
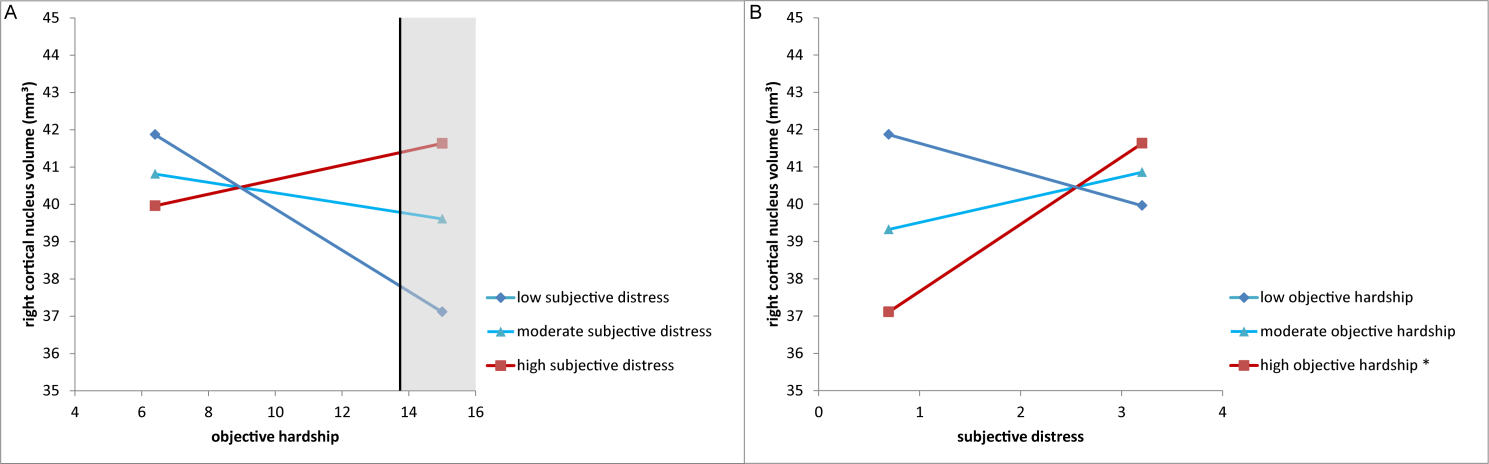
**

**Figure S5**. Interaction effects (p = 0.049, q = 0.686) between maternal objective hardship and subjective distress on the right cortical nucleus volume in young adult offspring, controlling for sex. A. When maternal objective hardship was equal to or larger than 13.74, higher maternal subjective distress was associated with larger right cortical nucleus volume in young adult offspring. The region of significance (p < 0.05) is represented by the vertical line and the gray shading. The dark blue line represents low maternal subjective distress; the bright blue line represents moderate maternal subjective distress, and the red line represents high maternal subjective distress. Low, moderate and high maternal subjective distress lines are represented at the 16th, 50th and 84th sample percentiles of maternal subjective distress scores, respectively, which were at a log-transformed score of 0.69, 2.08 and 3.20 (original score of 0.99, 7.00 and 23.53), respectively. B. The dark blue line represents low maternal objective hardship; the bright blue line represents moderate maternal objective hardship, and the red line represents high maternal objective hardship. Low, moderate and high maternal objective hardship lines are represented at the 16th, 50th and 84th sample percentiles of maternal objective hardship scores, respectively, which were at score of 6.40, 11.00 and 15.00, respectively. ^*^ p < 0.05

**
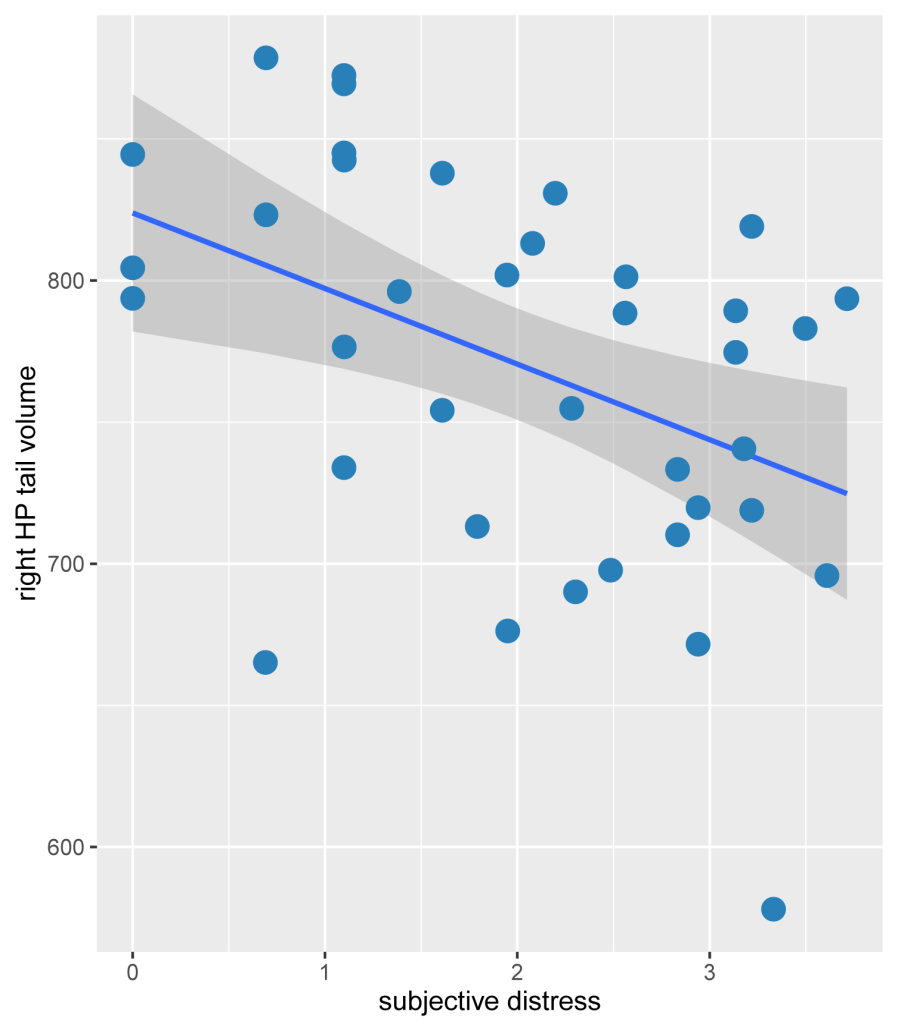
**

**Figure S6.** The association (beta = -0.437, p = 0.006, q = 0.234) between maternal subjective distress and right HP tail volume, controlling for sex. The blue regression lines represent associations between maternal subjective distress and right HP tail volume, controlling for sex. The gray shadings represent the 95% confidence interval.


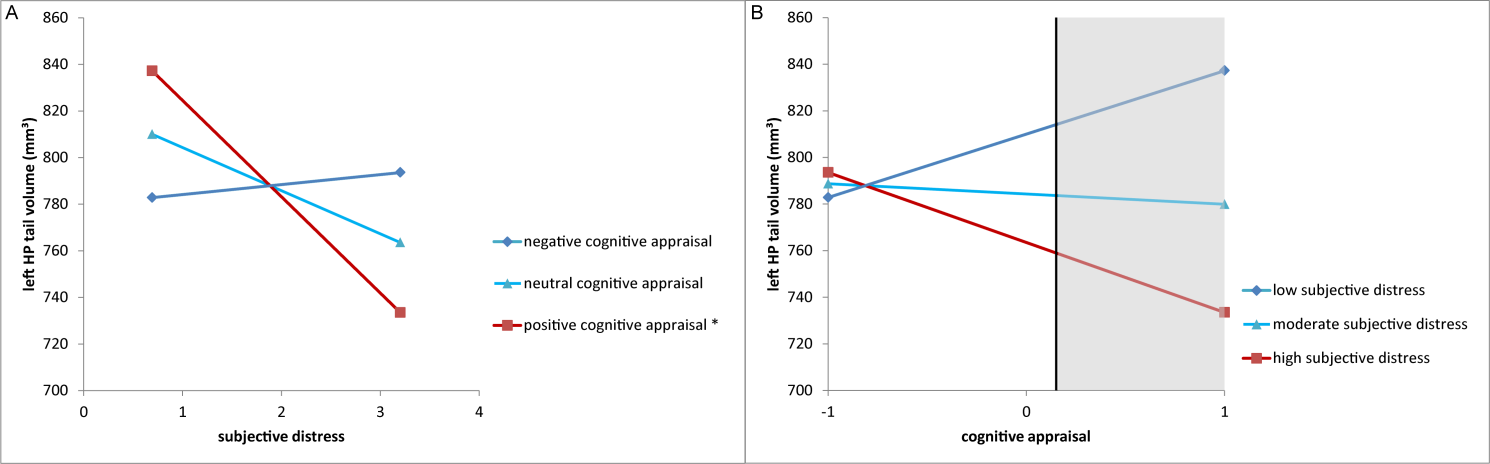


**Figure S7**. Interaction effects (p = 0.046, q = 0.322) between maternal subjective distress and cognitive appraisal on the left HP tail volume in young adult offspring, controlling for sex. A. The dark blue line represents negative maternal cognitive appraisal; the bright blue line represents neutral maternal cognitive appraisal, and the red line represents positive maternal cognitive appraisal. Negative, neutral and positive maternal cognitive appraisal lines are represented at the 16th, 50th and 84th sample percentiles of maternal cognitive appraisal scores, respectively, which were at score of -1, 0 and 1, respectively. ^*^ p < 0.05. B. When mothers reported positive cognitive appraisal, lower maternal subjective distress was associated with larger left HP tail volume in young adult offspring. The dark blue line represents low maternal subjective distress; the bright blue line represents moderate maternal subjective distress, and the red line represents high maternal subjective distress. Low, moderate and high maternal subjective distress lines are represented at the 16th, 50th and 84th sample percentiles of maternal subjective distress scores, respectively, which were at a log-transformed score of 0.69, 2.08 and 3.20 (original score of 0.99, 7.00 and 23.53), respectively. The region of significance (p < 0.05) is represented by the vertical line and the gray shading.


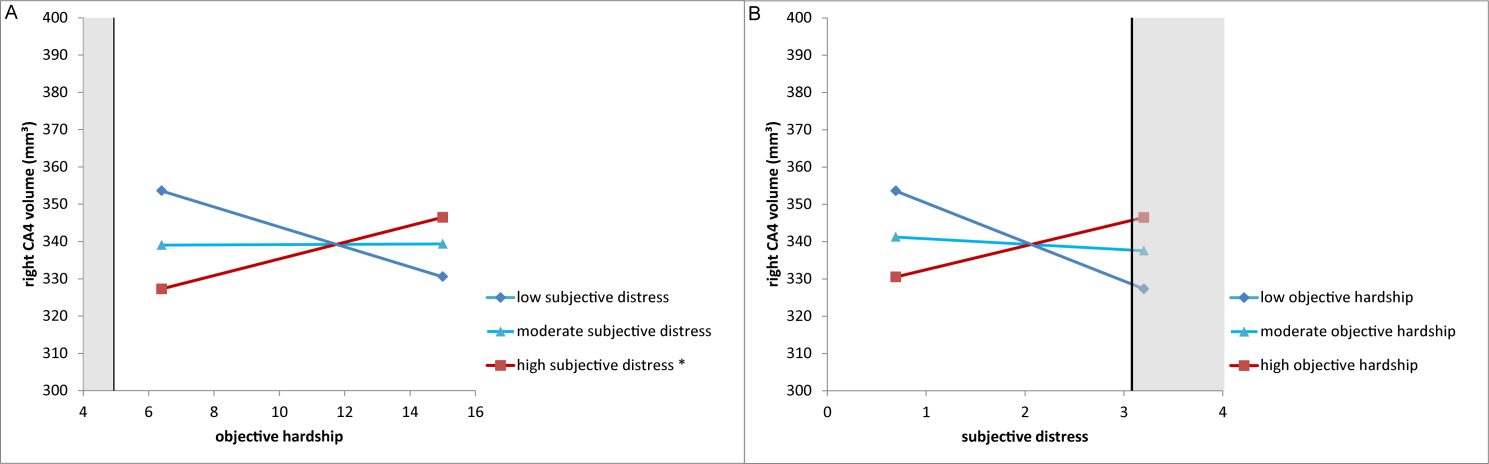


**Figure S8**. Interaction effects (p = 0.049, q = 0.322) between maternal objective hardship and subjective distress on the right CA4 volume in young adult offspring, controlling for sex. A. When maternal objective hardship was equal to or less than 4.92, lower maternal subjective distress was associated with larger right CA4 volume in young adult offspring. B. When maternal subjective distress was equal to or larger than a log-transformed value of 3.08 (original scale value of 20.76), higher maternal objective hardship was associated with larger right CA4 volume in young adult offspring. The dark blue line represents low maternal subjective distress or low maternal objective hardship; the bright blue line represents moderate maternal subjective distress or moderate maternal objective hardship, and the red line represents high maternal subjective distress or high maternal objective hardship. Low, moderate and high maternal subjective distress lines are represented at the 16th, 50th and 84th sample percentiles of maternal subjective distress scores, respectively, which were at a log-transformed score of 0.69, 2.08 and 3.20 (original score of 0.99, 7.00 and 23.53), respectively. Low, moderate and high maternal objective hardship lines are represented at the 16th, 50th and 84th sample percentiles of maternal objective hardship scores, respectively, which were at score of 6.40, 11.00 and 15.00, respectively. The regions of significance (p < 0.05) are represented by the vertical line and the gray shading. ^*^ p < 0.05.


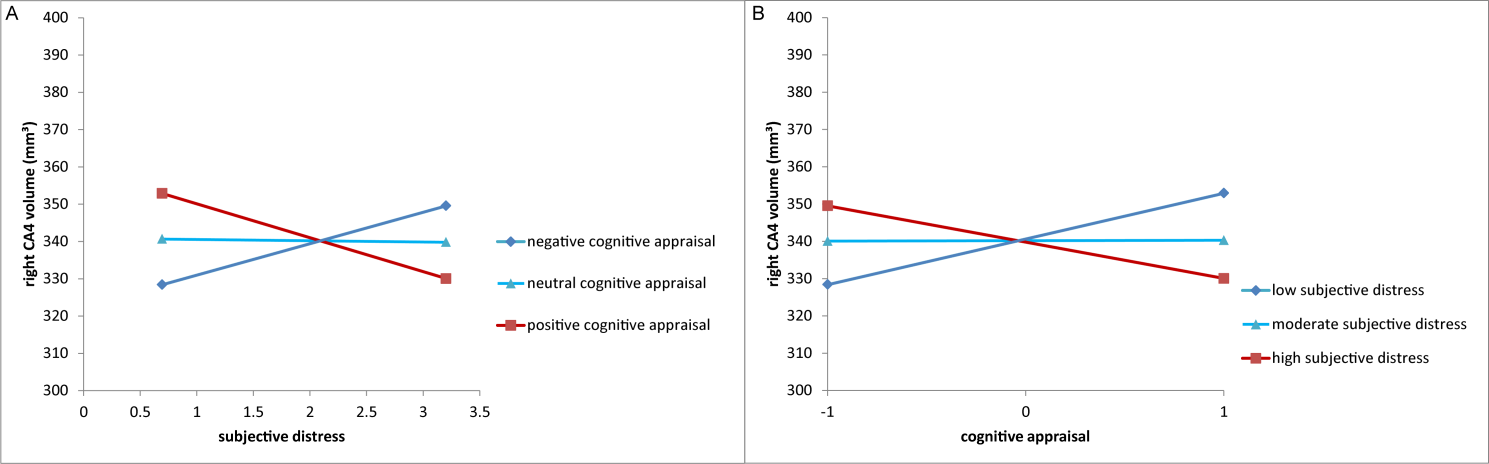


**Figure S9**. Interaction effects (p = 0.034, q = 0.322) between maternal subjective distress and cognitive appraisal on right CA4 volume in young adult offspring, controlling for sex. A. The dark blue line represents negative maternal cognitive appraisal; the bright blue line represents neutral maternal cognitive appraisal, and the red line represents positive maternal cognitive appraisal. Negative, neutral and positive cognitive appraisal lines are represented at the 16th, 50th and 84th sample percentiles of maternal cognitive appraisal scores, respectively, which were at score of -1, 0 and 1, respectively. B. The dark blue line represents low maternal subjective distress; the bright blue line represents moderate maternal subjective distress, and the red line represents high maternal subjective distress. Low, moderate and high maternal subjective distress lines are represented at the 16th, 50th and 84th sample percentiles of maternal subjective distress scores, respectively, which were at a log-transformed score of 0.69, 2.08 and 3.20 (original score of 0.99, 7.00 and 23.53), respectively.


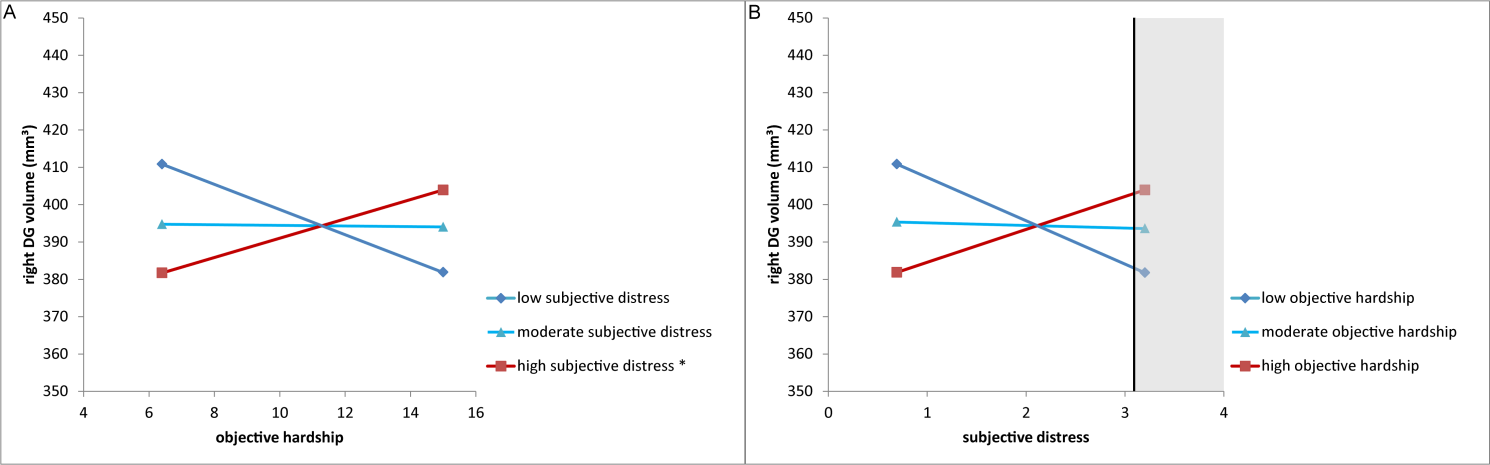


**Figure S10**. Interaction effects (p = 0.041, q = 0.322) between maternal objective hardship and subjective distress on the right DG volume in young adult offspring, controlling for sex. A. The dark blue line represents low maternal subjective distress; the bright blue line represents moderate maternal subjective distress, and the red line represents high maternal subjective distress. Low, moderate and high maternal subjective distress lines are represented at the 16th, 50th and 84th sample percentiles of maternal subjective distress scores, respectively, which were at a log-transformed score of 0.69, 2.08 and 3.20 (original score of 0.99, 7.00 and 23.53), respectively. B. When maternal subjective distress was equal to or larger than a log-transformed value of 3.09 (original scale value of 20.98), higher maternal objective hardship was associated with larger right DG volume in young adult offspring. The dark blue line represents low maternal objective hardship; the bright blue line represents moderate maternal objective hardship, and the red line represents high maternal objective hardship. Low, moderate and high maternal objective hardship lines are represented at the 16th, 50th and 84th sample percentiles of maternal objective hardship scores, respectively, which were at score of 6.40, 11.00 and 15.00, respectively. The region of significance (p < 0.05) is represented by the vertical line and the gray shading. ^*^ p < 0.05.

**
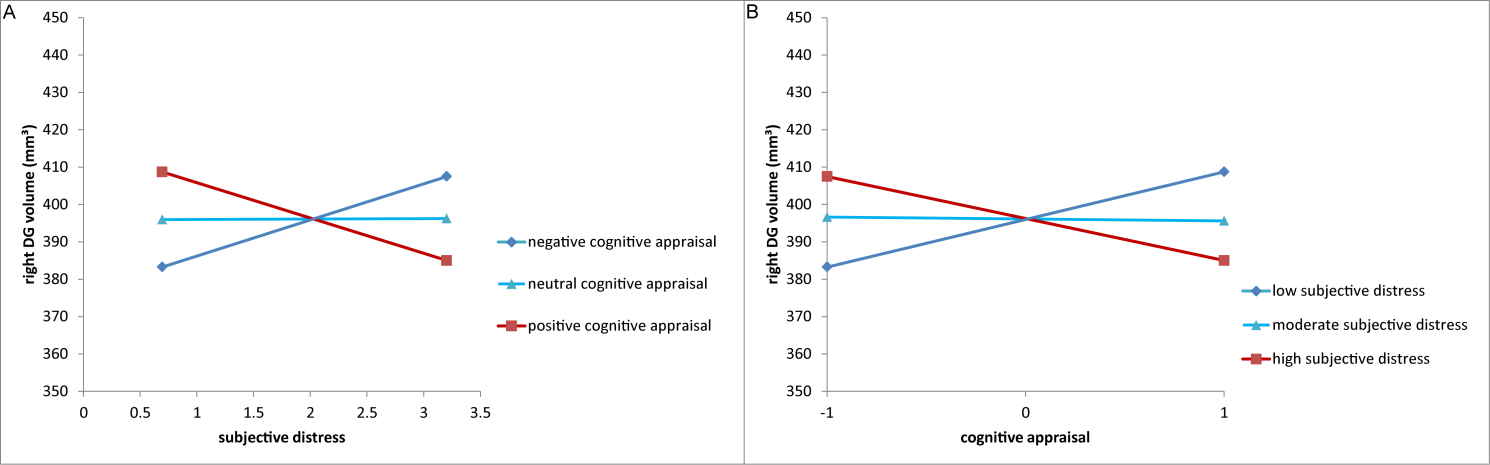
**

**Figure S11.** Interaction effects (p = 0.049, q = 0.322) between maternal subjective distress and cognitive appraisal on the right DG volume in young adult offspring, controlling for sex. A. The dark blue line represents negative maternal cognitive appraisal; the bright blue line represents neutral maternal cognitive appraisal, and the red line represents positive maternal cognitive appraisal. Negative, neutral and positive cognitive appraisal lines are represented at the 16th, 50th and 84th sample percentiles of maternal cognitive appraisal scores, respectively, which were at score of -1, 0 and 1, respectively. B. The dark blue line represents low maternal subjective distress; the bright blue line represents moderate maternal subjective distress, and the red line represents high maternal subjective distress. Low, moderate and high subjective distress lines are represented at the 16th, 50th and 84th sample percentiles of maternal subjective distress scores, respectively, which were at a log-transformed score of 0.69, 2.08 and 3.20 (original score of 0.99, 7.00 and 23.53), respectively.

**Table S4.** Ice Storm participants had lower amygdala nuclei-prefrontal rs-FC and lower hippocampal subfield-prefrontal rs-FC compared to controls.

| Regions | Beta | T score | p | q |
| --- | --- | --- | --- | --- |
| **Amygdala nuclei** | | | | |
| Seed: Right lateral nucleus | | | | |
| Left frontal pole | -0.19 | -3.74 | 0.000312 | 0.004994 |
| Seed: Right basal nucleus | | | | |
| Left superior frontal | -0.17 | -3.34 | 0.001177 | 0.009569 |
| Left caudal middle frontal | -0.19 | -3.34 | 0.001196 | 0.009569 |
| Seed: Right accessory basal nucleus | | | | |
| Left superior frontal | -0.18 | -3.03 | 0.003104 | 0.033635 |
| Right frontal pole | -0.15 | -2.93 | 0.004204 | 0.033635 |
| Seed: Right paralaminar nucleus | | | | |
| Left superior frontal | -0.20 | -3.78 | 0.000274 | 0.004378 |
| Right superior frontal | -0.18 | -3.47 | 0.000769 | 0.006050 |
| Left caudal middle frontal | -0.20 | -3.35 | 0.001134 | 0.006050 |
| Right caudal middle frontal | -0.12 | -2.55 | 0.012494 | 0.049966 |
| **Hippocampal subfields** |  |  |  |  |
| Seed: Left parasubiculum |  |  |  |  |
| Left superior frontal | -0.17 | -3.05 | 0.002949 | 0.038858 |
| Right superior frontal | -0.17 | -2.84 | 0.005548 | 0.038858 |
| Right frontal pole | -0.13 | -2.74 | 0.007286 | 0.038858 |


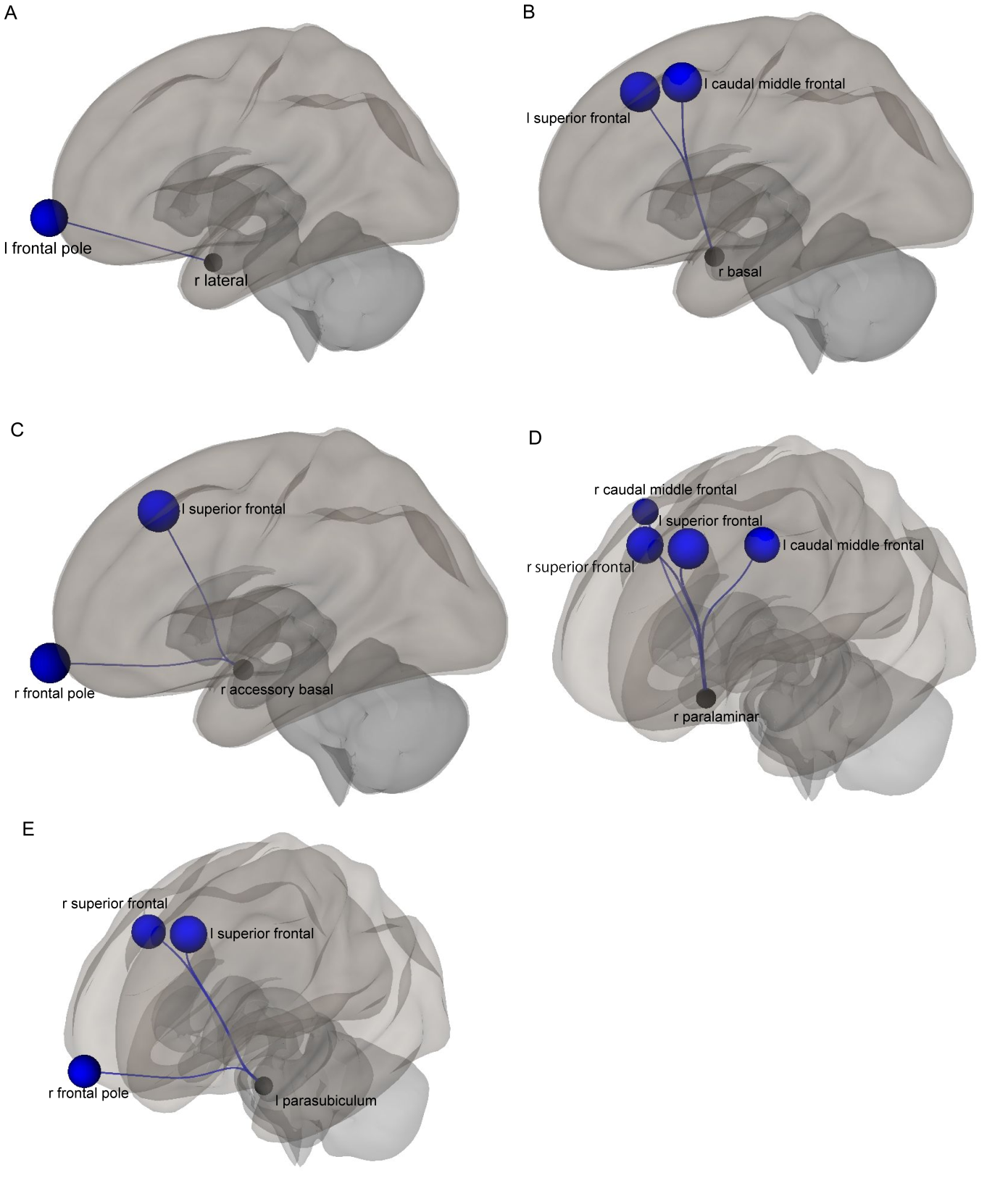


**Figure S12**. Compared to controls, Ice Storm participants had seed-to-seed rs-FC between the amygdala nuclei and prefrontal subregions, between the hippocampal subfields and prefrontal subregions. **A** rs-FC between the right lateral nucleus and the left frontal pole. **B** rs-FC between the right basal nucleus and the left superior frontal and the left caudal middle frontal. **C** rs-FC between the right accessary basal nucleus and the right frontal pole, the left superior frontal. **D** rs-FC between the right paralaminar nucleus and bilateral superior frontal and bilateral caudal middle frontal. **E** rs-FC between the left parasubiculum and bilateral superior frontal and the right frontal pole. The dark gray nodes represent the source seed, and the blue nodes represent the target seed. The nodes are linked with blue lines, which represent lower rs-FC in Ice Storm participants than that in controls.


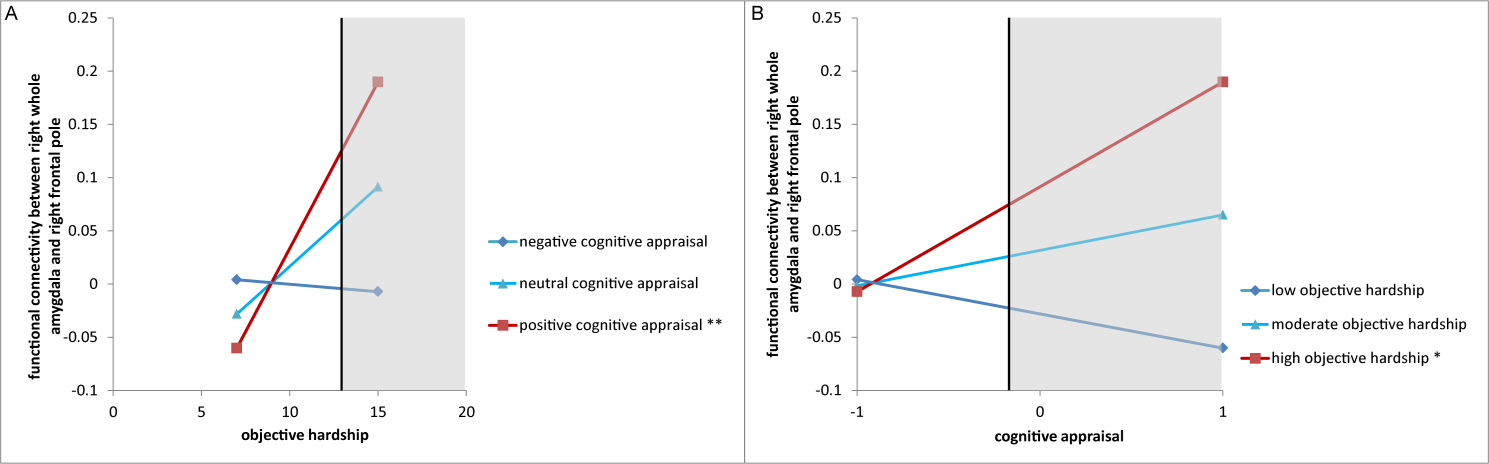


**Figure S13**. Interaction effects (p = 0.022, q = 0.264) between maternal objective hardship and cognitive appraisal on functional connectivity between right whole amygdala and right frontal pole in young adult offspring, controlling for sex. A. When maternal objective hardship was equal to or larger than 12.94, more negative maternal cognitive appraisal was associated with lower functional connectivity between the right whole amygdala and the right frontal pole in young adult offspring. The dark blue line represents negative maternal cognitive appraisal; the bright blue line represents neutral maternal cognitive appraisal, and the red line represents positive maternal cognitive appraisal. Negative, neutral and positive maternal cognitive appraisal lines are represented at the 16th, 50th and 84th sample percentiles of maternal cognitive appraisal scores, respectively, which were at score of -1, 0 and 1, respectively. B. When maternal cognitive appraisal was neutral or positive, lower maternal objective hardship was associated with lower functional connectivity between the right whole amygdala and the right frontal pole in young adult offspring. The dark blue line represents low maternal objective hardship; the bright blue line represents moderate maternal objective hardship, and the red line represents high maternal objective hardship. Low, moderate and high maternal objective hardship lines are represented at the 16th, 50th and 84th sample percentiles of maternal objective hardship scores, respectively, which were at a log-transformed score of 7, 11 and 15, respectively. The regions of significance (p < 0.05) are represented by the vertical line and the gray shading. ^**^ p < 0.01; ^*^ p < 0.05.


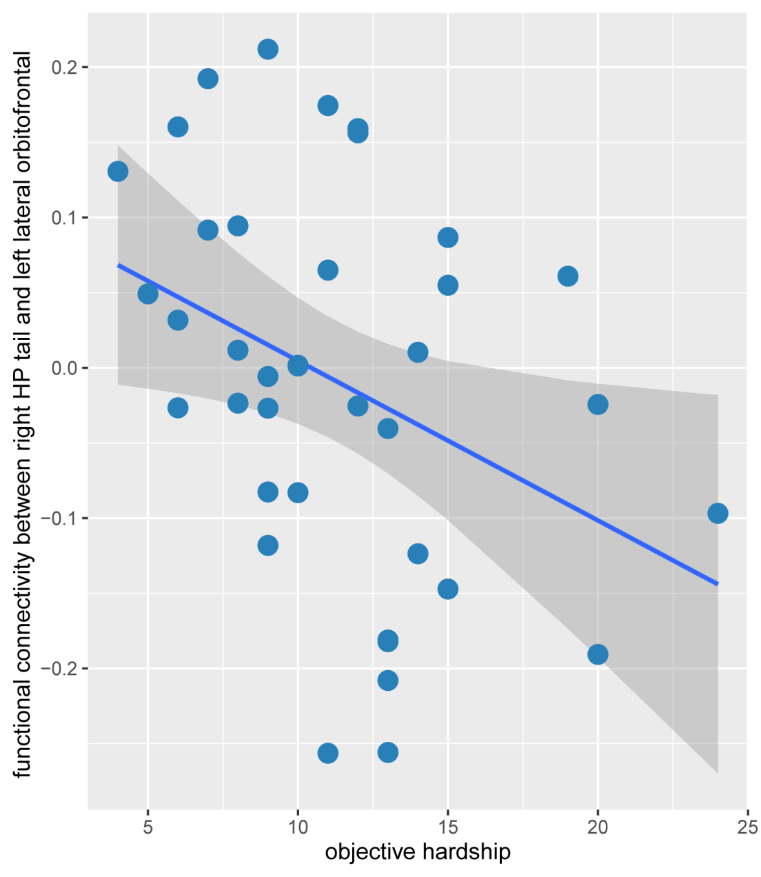


**Figure S14**. The association (beta = -0.361, p = 0.028, q = 0.084) between maternal objective hardship and functional connectivity from the right HP tail to the left lateral orbitofrontal with controlling for sex. The blue regression lines represent associations between maternal objective hardship and functional connectivity between the right HP tail and the left lateral orbitofrontal with controlling for sex. The gray shadings represent the 95% confidence interval.
